# Label-efficient seedling perception via semi-supervised learning and counterfactual spatiotemporal feature rectification

**DOI:** 10.1016/j.plaphe.2026.100278

**Published:** 2026-09-08

**Authors:** Yaoyao Nie, Huimin Ma, Xiaoshen Wang, Pengfei Zhang, Yi Wu, Xiaohong Li, Lichuan Gu

**Affiliations:** a School of Artificial Intelligence, Anhui Agricultural University, Hefei, 230036, China; b Anhui Province Key Laboratory of Smart Agricultural Technology and Equipment, Hefei, 230036, China; c Institute of Intelligent Machines, HFIPS, Chinese Academy of Sciences, Hefei, 230031, China; d Henan Zhongke Science and Technology Achievement Transfer Center, Zhengzhou, 450008, China; e College of Electronics and Electrical Engineering, Anhui Agricultural University, Hefei, 230036, China; f University of Science and Technology of China, Hefei, 230026, China

**Keywords:** Missing-seedling gap inference, Dual-stream anchor refinement, Masked trajectory inpainting, Counterfactual spatiotemporal rectification, Dual-scale inertial metric

## Abstract

In precision agriculture, assessing missing seedlings is essential for variable-rate reseeding and yield estimation. However, missing plants represent a semantic absence rather than explicit visual targets, making them difficult to define using conventional bounding-box annotations. To address this challenge, we propose a video-based, detection-driven gap inference framework for UAV wheat seedling phenotyping. Specifically, a semi-supervised Dual-Stream Anchor-Refinement Tracking (DART) mechanism is introduced to generate reliable pseudo-labels for visible seedlings from sparse temporal anchors, reducing dependence on dense manual annotations. To address visual fragmentation and weak target responses, a Masked Trajectory Inpainting Network (MTI-Net) combines object-aware masking with history-guided feature reconstruction to recover incomplete seedling representations using temporal context. The refined visible-seedling detections are then converted into agronomic gap indicators through a Dual-Scale Inertial Metric (DS-IM), which incorporates mechanical-sowing kinematic regularities and local growth-space constraints. Evaluations on the UAV-WheatSeedling dataset demonstrate positive mean gains when the proposed modules are embedded into RT-DETR and multiple YOLO-family backbones. When integrated with YOLO12-S, the method achieves 92.92 ± 0.17% mAP@0.50, 69.33 ± 0.22% mAP@[0.5:0.95], and 86.59 ± 0.41% Recall. Under 50% sparse-anchor supervision, the proposed framework approaches the 100% sparse-anchor YOLO12 baseline in mAP@[0.5:0.95], while achieving higher mean Recall. For downstream gap inference, DS-IM achieves an F1-score of 84.53% and a frame-level count MAE of 0.229 gaps/frame. This study provides a data-efficient and interpretable framework for low-altitude UAV-based early seedling assessment.

## 1 Introduction

In the context of precision agriculture, early-stage crop stand establishment is a critical determinant of final yield potential [1]. In practical field scenarios, however, emergence rates often exhibit significant spatial heterogeneity due to inherent sowing instabilities and the spatio-temporal variability of soil physical properties [2]. Specifically, emergence deficits, which manifest as discontinuous segments within crop rows, lead to the inefficient utilization of light and water resources, causing irreversible yield losses [3]. The agronomic necessity of such early assessment has been extensively validated across major cropping systems [4]. Therefore, rapid and accurate field-scale detection of these deficits is agronomically essential for guiding variable-rate reseeding and achieving precise yield forecasting [5].

Low-altitude Unmanned Aerial Vehicle (UAV) remote sensing has emerged as a high-throughput alternative to labor-intensive manual sampling [6,7]. Although recent deep learning advances have improved seedling counting from static UAV imagery [8], robust detection in unstructured fields remains challenging [9]. Early-stage seedlings occupy small image regions whose weak feature responses can be attenuated during progressive network downsampling [10] or confounded by heterogeneous soil backgrounds [11]. Multi-scale feature fusion can help preserve fine spatial details [[12], [13], [14]], but reliable representation of these minute targets remains difficult across variable field conditions [15].

Beyond target scale, the fragmented morphology of early-stage wheat seedlings introduces an additional challenge. UAV-based small-object detection already operates with limited effective spatial information [[16], [17], [18]]; under nadir imaging, thin and diverging leaves may appear as several disconnected image regions rather than a compact visual target. Single-frame models may therefore struggle to associate these dispersed responses into a coherent seedling representation, particularly under low contrast, motion blur, and background interference. These characteristics motivate the use of temporal evidence to associate fragmented responses across adjacent frames.

Video-based deep learning offers a promising avenue by incorporating temporal information to resolve these ambiguities [[19], [20], [21]]. Teacher–student formulations have also been explored for semi-supervised video learning. For example, Stable Mean Teacher improves video pseudo-label learning through a student–teacher framework and temporal-consistency constraints in semi-supervised video action detection [22]. However, directly adapting general Video Object Detection (VOD) frameworks or emerging self-supervised paradigms [[23], [24], [25]] to agricultural phenotyping remains challenging. Two characteristics are particularly relevant. First, some widely used VOD methods employ explicit optical flow to model relatively large rigid displacements [23,26]. In contrast, early-stage crops are approximately stationary in the field coordinate system, and their visual variations are often dominated by high-frequency, non-rigid perturbations such as leaf fluttering. This difference can weaken temporal correspondence under transient motion blur or aerodynamic turbulence [[27], [28], [29]]. Second, while Masked Autoencoders enhance robustness in general vision [[30], [31], [32], [33]], random masking may disproportionately remove the already limited feature responses of minute seedlings. Without object-aware masking and target-aligned temporal constraints, recovering seedling-related representations across spatio-temporal dimensions remains difficult [[34], [35], [36]]. This motivates a trajectory-oriented reconstruction formulation: feature corruption is restricted to seedling-object regions, while missing responses are recovered from temporally corresponding historical features.

Furthermore, formally defining missing seedlings poses a unique semantic challenge. Unlike physical objects that possess distinct morphological boundaries, a gap represents a semantic absence, a disruption in geometric continuity rather than a feature-rich target [37,38]. Consequently, standard supervised learning, which relies on explicit positive annotations, struggles to characterize these negative regions. An Efficient Teacher-based semi-supervised pipeline has recently been applied to UAV monitoring of early-stage maize seedlings, where seedlings and missing seedlings were directly modeled as detection classes [39]. Nevertheless, the absence of a plant provides limited localized appearance evidence for reliable bounding-box regression [40]. Advanced crop row detection frameworks have successfully refined macro-structural field representations [41]. However, these frameworks are designed primarily for row-level structural continuity rather than explicit local missing-seedling gap inference.

To address these limitations, we formulate missing-seedling assessment as detection-driven gap inference rather than direct missing-object detection. Since a gap lacks intrinsic visual texture and object boundaries, its existence cannot be reliably determined from localized appearance alone. Instead, potential gaps are inferred from the spatial discontinuity of visible seedlings under mechanical-sowing and agronomic constraints. Guided by this formulation, this study proposes a comprehensive video-based framework for label-efficient wheat seedling perception and missing-seedling gap inference. The framework couples visible-seedling detection with geometry- and agronomy-constrained gap inference and consists of three core components:

• *Dual-Stream Anchor-Refinement Tracking (DART):* To achieve label-efficient perception by alleviating the annotation costs associated with dense and repetitive agricultural patterns [42], we propose DART as a semi-supervised pseudo-label refinement framework. To reduce the error accumulation of causal recursive tracking paradigms [[43], [44], [45]], DART employs non-causal bidirectional inference between sparse temporal anchors. Combined with uncertainty quantification [46] and temporal-distance-aware Bayesian fusion, it refines accepted tracking predictions into reliable visible-seedling supervision [47,48].
• *Masked Trajectory Inpainting Network (MTI-Net):* To enhance temporal feature representations, we introduce MTI-Net as an object-aware and history-guided masked feature reconstruction module. Unlike standard masked autoencoder approaches based on spatially random masking [31], MTI-Net selectively corrupts seedling-object regions in the current-frame feature map and reconstructs the missing responses from temporally corresponding historical features. This object-aware reconstruction along temporal feature trajectories, analogous to motion-guided spatiotemporal propagation [49], improves temporal feature continuity for fragmented seedlings.
• *Dual-Scale Inertial Metric (DS-IM):* To translate visible-seedling detections into candidate gap locations and counts, we develop DS-IM as a geometry- and agronomy-constrained gap-inference procedure. It combines macro-scale row-periodicity reconstruction with micro-scale trajectory and growth-space verification to infer gaps from the spatial distribution of visible seedlings.

## 2 Materials and methods

### 2.1 Data acquisition and specifications

To facilitate this study, we constructed a custom UAV-based video dataset, designated as UAV-WheatSeedling, to capture winter wheat during the critical emergence stage. Data were collected from two independent wheat fields within the same study region at the Anhui Academy of Agricultural Sciences, China (study-region center: 31.8877°N, 117.2460°E), from November to December 2024. Aerial views of Field 1 and Field 2 are shown in Fig. 1A–B, while their geographic locations within the study region are indicated in Fig. 1D. The two fields differed in wheat cultivar, planting density, local field environment, and soil/background appearance. Data collection further covered four representative imaging conditions: sunny-dry, sunny-wet, overcast-dry, and overcast-wet, as illustrated in Fig. 1C. To resolve fine seedling morphology, a DJI Air 2S drone (1-inch CMOS) captured UHD video (3840 × 2160 at 30 fps). According to the session-calibrated onboard telemetry, the flight height varied dynamically within a low-altitude range of 1.5–5.0 m, with a median of 2.1 m and a 99th percentile of 3.1 m. The gimbal was maintained at a nadir view (−90°), as schematically illustrated in Fig. 1E. This low-altitude nadir-view configuration was selected to retain sufficient spatial detail for early-stage seedlings and to provide a stable spatial reference for stand-gap analysis.

**Fig. 1:**
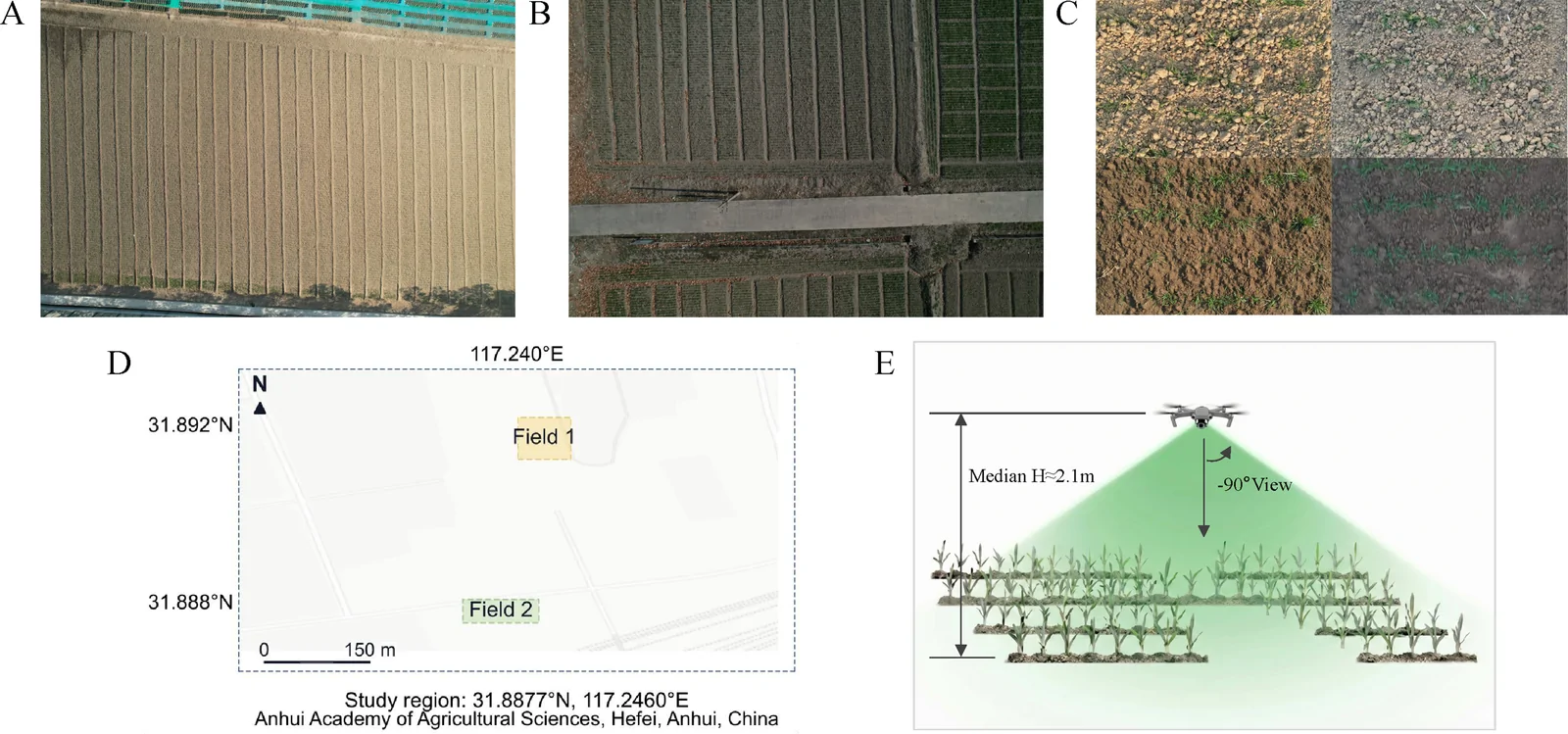
Overview of the UAV-WheatSeedling dataset. (A–B) Aerial views of Field 1 and Field 2. (C) Representative imaging conditions: sunny-dry, sunny-wet, overcast-dry, and overcast-wet. (D) Geographic locations of the two experimental fields within the study region. (E) Schematic illustration of nadir-view acquisition under dynamically varying low-altitude conditions (*H* = 1.5–5.0 m; median *H* = 2.1 m).

The finalized dataset comprises 35 sequences (∼189,000 frames). The sequences were partitioned at the sequence level into training, validation, and test sets accounting for approximately 70%, 17%, and 13% of the total frames, respectively. No sequence was shared across subsets. Manual bounding-box annotations were provided on sparsely sampled keyframes, generally at intervals of approximately 120 frames. The complete set of sparsely annotated anchors is hereafter referred to as 100% sparse-anchor supervision in the label-efficiency experiments, with lower percentages indicating proportional subsampling of this set. The 10%, 25%, and 50% sparse-anchor ratios were applied only to the training anchor pool, while the validation and test annotations were kept fixed across the label-efficiency experiments.

### 2.2 System overview

The proposed framework comprises three stages: DART-based supervisory generation, MTI-Net-based spatiotemporal seedling perception, and DS-IM-based gap inference, as illustrated in Fig. 2a. Together, they form a modular workflow from sparse-anchor annotations to visible-seedling detection and missing-seedling gap inference. During training, DART propagates seedling states between sparsely annotated anchors and refines reliable intermediate predictions into pseudo-labels, which are subsequently used to supervise the detector equipped with MTI-Net. MTI-Net enhances weak or fragmented seedling representations by retrieving historical temporal features, while its auxiliary branch performs object-aware masked reconstruction during training. The detector and MTI-Net are optimized using the detection and reconstruction objectives, whereas DART serves as the preceding supervisory-generation stage and is not optimized by these objectives. During inference, the auxiliary reconstruction branch is removed, and DS-IM converts the resulting visible-seedling detections into candidate gap locations and counts using mechanical-sowing kinematic regularities and local growth-space constraints. The principal mathematical symbols used throughout the framework are summarized in Table S7.

**Fig. 2:**
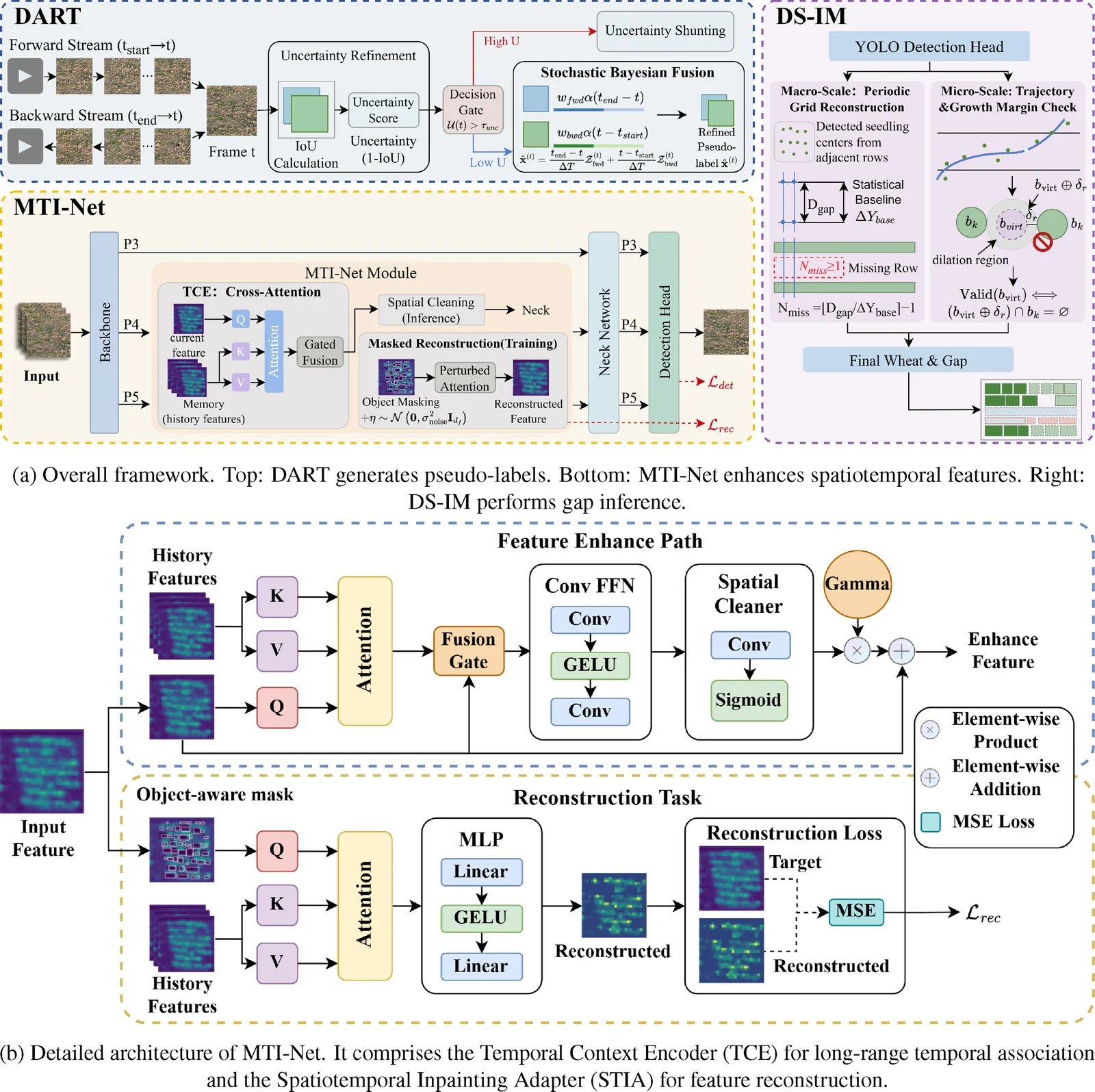
The proposed system framework. (a) Overall pipeline. (b) Architecture of MTI-Net.

### 2.3 Semi-supervised DART framework

To address the high labor costs and subjectivity inherent in UAV video annotation, we propose the Dual-Stream Anchor-Refinement Tracking (DART) framework. Traditional unidirectional recursive tracking often suffers from error accumulation. DART instead defines sparse ground-truth frames as temporal anchors and establishes a non-causal, closed-loop inference system. By integrating a dual-stream uncertainty quantification module with a stochastic Bayesian smoothing mechanism, the framework dynamically shunts training samples to generate refined pseudo-labels from noisy tracking observations.

#### 2.3.1 Bidirectional inference and uncertainty-aware shunting

To effectively capture long-term temporal dependencies within video sequences and precisely evaluate the reliability of unlabeled intermediate frames, DART establishes a bidirectional inference mechanism. For any ground-truth interval (*t*_*start*_, *t*_*end*_) defined by temporal anchors, the system concurrently executes a forward inference stream $\left(Z_{\mathrm{fwd}}\right)$ and a backward inference stream $\left(Z_{\mathrm{bwd}}\right)$:

• **Forward Inference Stream** $\left(Z_{\mathrm{fwd}}\right)$: propagates from the past anchor $I_{t_{\mathrm{start}}}$ strictly forward along the time axis to infer future states.
• **Backward Inference Stream** $\left(Z_{\mathrm{bwd}}\right)$: propagates from the future anchor $I_{t_{\mathrm{end}}}$ recursively backward to infer past states.

For any intermediate frame *t* within the interval, the forward and backward streams yield two direction-specific bounding-box predictions, $Z_{\mathrm{fwd}}^{\left(t\right)}$ and $Z_{\mathrm{bwd}}^{\left(t\right)}$. Their spatial disagreement is used as an observable proxy for tracking reliability in the absence of dense supervisory signals [46,50]. Consistent bidirectional predictions generally indicate stable tracking, whereas a large discrepancy suggests an increased risk of tracking failure under conditions such as occlusion, motion blur, or non-rigid appearance variation:

(1) $$U\left(t\right)=1-\frac{1}{N_{t}}\overset{N_{t}}{\underset{i=1}{\sum }}\text{IoU}\left(Z_{\mathrm{fwd,i}}^{\left(t\right)},Z_{\mathrm{bwd,i}}^{\left(t\right)}\right)$$

where $Z_{\mathrm{fwd,i}}^{\left(t\right)}$ and $Z_{\mathrm{bwd,i}}^{\left(t\right)}$ represent the bounding box predictions for the *i*-th instance from the forward and backward passes, respectively, and *N*_*t*_ is the number of tracked instances. IoU(⋅) denotes the Intersection over Union metric.

Based on this measure, DART separates high-risk observations from samples retained for automatic pseudo-label refinement:

(2) $$\text{Action}\;\left(t\right)=\left\{\begin{array}{ll} \text{Pseudo}-\text{label Exclusion}, & U\left(t\right)>\tau_{\mathrm{unc}},\\ \text{Bayesian Refinement}, & U\left(t\right)\leq \tau_{\mathrm{unc}}, \end{array}\right)$$

where *τ*_*unc*_ controls the accepted level of bidirectional disagreement. Samples exceeding this threshold are excluded from pseudo-label generation, whereas the retained low-disagreement samples proceed to temporal-distance-aware Bayesian refinement.

#### 2.3.2 Wiener-type temporal-distance-aware Bayesian Refinement

For the low-disagreement samples retained after uncertainty shunting, DART applies a temporal-distance-aware Bayesian fusion strategy. Seedlings remain approximately stationary in the field coordinate system, so we do not model their physical motion as a stochastic process. Instead, we model the accumulation of tracking error as predictions are propagated away from their corresponding annotated anchors [51]. Within the finite interval between two adjacent anchors, this accumulation is approximated using a first-order Wiener-type diffusion model, under which the error covariance increases with temporal propagation distance. This local approximation is applied only to samples retained within the low-disagreement refinement regime.

Let **x**^(*t*)^ denote the latent bounding-box state of a target at frame *t*. The forward stream propagates over a temporal interval Δ*t*_*fwd*_ = *t* − *t*_*start*_, whereas the backward stream propagates over Δ*t*_*bwd*_ = *t*_*end*_ − *t*. The corresponding direction-specific observations are modeled as

(3) $$\begin{array}{l} Z_{\mathrm{fwd}}^{\left(t\right)}=x^{\left(t\right)}+\epsilon_{\mathrm{fwd}}^{\left(t\right)},\\ Z_{\mathrm{bwd}}^{\left(t\right)}=x^{\left(t\right)}+\epsilon_{\mathrm{bwd}}^{\left(t\right)}, \end{array}$$

where $\epsilon_{\mathrm{fwd}}^{\left(t\right)}$ and $\epsilon_{\mathrm{bwd}}^{\left(t\right)}$ denote the tracking errors accumulated from the two annotated anchors.

Under the local diffusion approximation, the direction-specific tracking errors are modeled as

(4) $$\begin{array}{l} \epsilon_{\mathrm{fwd}}^{\left(t\right)}∼N\;\left(0,\alpha \Delta t_{\mathrm{fwd}}I_{d_{x}}\right),\\ \epsilon_{\mathrm{bwd}}^{\left(t\right)}∼N\;\left(0,\alpha \Delta t_{\mathrm{bwd}}I_{d_{x}}\right), \end{array}$$

where *α* is the local diffusion-scale coefficient, *d*_*x*_ is the dimensionality of the tracked state, and $I_{d_{x}}$ is the corresponding identity matrix. A shared diffusion scale is adopted as a parsimonious approximation because the two streams use the same tracking configuration within the same anchor interval. Since only their relative variances are required for fusion, the common coefficient *α* cancels from the final weighting rule.

For a tractable closed-form estimate, the two direction-specific tracking errors are approximated as conditionally independent given the latent state. Under a locally non-informative prior, the maximum a posteriori estimate reduces to the precision-weighted combination

(5) $${\hat{x}}^{\left(t\right)}=\frac{\sigma_{\mathrm{bwd}}^{2}\cdot Z_{\mathrm{fwd}}^{\left(t\right)}+\sigma_{\mathrm{fwd}}^{2}\cdot Z_{\mathrm{bwd}}^{\left(t\right)}}{\sigma_{\mathrm{fwd}}^{2}+\sigma_{\mathrm{bwd}}^{2}}$$

where

(6) $$\sigma_{\mathrm{fwd}}^{2}=\alpha \Delta t_{\mathrm{fwd}},\;\sigma_{\mathrm{bwd}}^{2}=\alpha \Delta t_{\mathrm{bwd}}.$$

Substituting Eq. (6) into Eq. (5) yields

(7) $${\hat{x}}^{\left(t\right)}=\frac{\Delta t_{\mathrm{bwd}}}{\Delta T}Z_{\mathrm{fwd}}^{\left(t\right)}+\frac{\Delta t_{\mathrm{fwd}}}{\Delta T}Z_{\mathrm{bwd}}^{\left(t\right)},\;\Delta T=t_{\mathrm{end}}-t_{\mathrm{start}}.$$

Accordingly, the contribution of each stream decreases linearly with its propagation distance from the corresponding anchor. A prediction closer to its originating anchor therefore receives a larger fusion weight, reducing the influence of errors accumulated over longer propagation paths. The resulting estimate ${\hat{x}}^{\left(t\right)}$ is used as the refined pseudo-label for the retained intermediate frame.

### 2.4 MTI-Net: temporal masked modeling for feature enhancement

DART alleviates supervision sparsity by generating refined pseudo-labels, whereas the underlying detector must still recover weak seedling representations from the video stream. In field environments, occlusion, motion blur, leaf motion, and heterogeneous backgrounds can fragment or suppress the already limited feature responses of small seedlings, increasing the risk of missed detections. To incorporate complementary temporal evidence directly into the feature representation, we introduce the Masked Trajectory Inpainting Network (MTI-Net), whose integration with the detector is illustrated in Fig. 2a.

MTI-Net performs counterfactual spatiotemporal feature rectification by coupling object-aware feature corruption with history-guided temporal retrieval. During training, selected seedling-object regions in the current-frame feature map are deliberately corrupted, and the network is encouraged to reconstruct their clean representations from temporally corresponding historical features. Following the general principle of self-supervised temporal reconstruction [52], MTI-Net reconstructs corrupted object-region features from their temporal context, directing the auxiliary objective toward weak or fragmented target regions rather than irrelevant background content. As detailed in Fig. 2b, MTI-Net comprises two cascaded modules. The Temporal Context Encoder (TCE) establishes temporal associations and retrieves historical features, whereas the Spatiotemporal Inpainting Adapter (STIA) combines inference-time spatial cleaning with training-time object-aware feature reconstruction.

#### 2.4.1 Temporal context encoder

Let $X\in R^{T\times C\times H\times W}$ denote the backbone feature sequence ending at the current frame *t*. TCE first applies a lightweight depthwise 3D convolution for local spatial encoding, together with a residual connection and a learnable temporal positional embedding:

(8) $$X_{\mathrm{loc}}=X+\text{Conv}3\text{D}\left(X\right)+P_{\mathrm{pos}}$$

where $P_{\mathrm{pos}}\in R^{T\times C\times 1\times 1}$ is broadcast across spatial dimensions.

Subsequently, TCE employs a target-oriented Cross-Attention mechanism rather than standard self-attention. Specifically, the current-frame feature $X_{\mathrm{loc}}^{\left(t\right)}$ serves as the Query (*Q*). The flattened historical features $X_{\mathrm{loc}}^{\left(1:t-1\right)}$ $\left(X_{\mathrm{hist}}\right)$ serve as Keys (*K*) and Values (*V*). This query-retrieval process aggregates relevant information from preceding frames to compensate for the degradation in the current frame:

(9) $$F_{\mathrm{attn}}^{\left(t\right)}=\text{Attention}\left(Q=X_{\mathrm{loc}}^{\left(t\right)},K=X_{\mathrm{hist}},V=X_{\mathrm{hist}}\right)$$

Unlike simple residual addition, we introduce a Gated Fusion mechanism to adaptively integrate the retrieved temporal information with the static visual semantics. The final temporally enhanced representation *F*_*fused*_ is formulated as:

(10) $$F_{\mathrm{fused}}^{\left(t\right)}=X_{\mathrm{loc}}^{\left(t\right)}+\sigma \left(G\left(\left[X_{\mathrm{loc}}^{\left(t\right)},F_{\mathrm{attn}}^{\left(t\right)}\right]\right)\right)⊙F_{\mathrm{attn}}^{\left(t\right)}$$

where *σ*(⋅) denotes the Sigmoid function, G represents a convolutional gating network, and ⊙ denotes element-wise multiplication. The fused representation $F_{\mathrm{fused}}^{\left(t\right)}$ is further refined by a lightweight ConvFFN with a zero-initialized residual scale, $F_{\mathrm{enh}}^{\left(t\right)}=F_{\mathrm{fused}}^{\left(t\right)}+\gamma_{\mathrm{ffn}}\text{ConvFFN}\left(F_{\mathrm{fused}}^{\left(t\right)}\right)$. STIA subsequently applies spatial cleaning to the enhanced feature along the inference path, while its auxiliary training path performs object-aware feature reconstruction.

#### 2.4.2 Spatiotemporal Inpainting Adapter

The STIA module operates through a dual-stream mechanism designed to reconcile inference stability with training robustness. To mitigate the ghosting artifacts introduced by temporal aggregation in unstructured fields, the Inference Stream employs a Spatial Cleaning mechanism. Unlike standard attention outputs, we impose a filtering step based on local feature statistics. Specifically, we compute the channel-wise average and maximum pooling of the enhanced feature $F_{\mathrm{enh}}^{\left(t\right)}$ to distinguish foreground target responses from background interference. These statistics are fed into a lightweight convolutional layer with a Sigmoid activation to generate a soft spatial mask $M_{\mathrm{clean}}\in {\left[0,1\right]}^{H\times W}$:

(11) $$M_{\mathrm{clean}}=\sigma \left({\text{Conv}}_{7\times 7}\left(\left[\text{Avg}\left(F_{\mathrm{enh}}^{\left(t\right)}\right);\text{Max}\left(F_{\mathrm{enh}}^{\left(t\right)}\right)\right]\right)\right)$$

where Avg and Max denote average pooling and max pooling, respectively. The final refined feature is obtained by element-wise filtration: $F_{\mathrm{final}}^{\left(t\right)}=F_{\mathrm{enh}}^{\left(t\right)}⊙M_{\mathrm{clean}}$. The module uses a global zero-gamma residual connection, $F_{\mathrm{out}}^{\left(t\right)}=F_{in}^{\left(t\right)}+\gamma \cdot F_{\mathrm{final}}^{\left(t\right)}$, where $F_{in}^{\left(t\right)}$ denotes the current-frame shortcut feature. This initialization allows the module to progressively learn to suppress background noise while preserving target integrity.

During training, the Auxiliary Stream introduces a feature-reconstruction objective that encourages the attention mechanism to learn robust temporal correspondences under feature degradation. Let $F_{\mathrm{cur}}^{\left(t\right)}$ denote the normalized current-frame feature used by the auxiliary reconstruction branch. Leveraging the unified label space, we construct an object-aware binary mask **M** from the seedling-object regions and inject Gaussian noise into the masked regions to simulate feature corruption:

(12) $${\tilde{F}}^{\left(t\right)}=F_{\mathrm{cur}}^{\left(t\right)}⊙\left(1-M\right)+\eta^{\left(t\right)}⊙M,$$

where $\eta^{\left(t\right)}∼N\;\left(0,\sigma_{\text{noise}}^{2}I_{d_{f}}\right)$ denotes independently sampled Gaussian feature noise with standard deviation *σ*_noise_. Here, *d*_*f*_ is the dimensionality of the vectorized feature representation, and $I_{d_{f}}$ is the corresponding identity matrix. The binary mask **M** has the same spatial support as $F_{\mathrm{cur}}^{\left(t\right)}$, with values of one in the selected seedling-object regions and zero elsewhere; ⊙ denotes element-wise multiplication.

The object-aware mask restricts feature corruption to seedling-related regions, preventing the auxiliary task from being dominated by irrelevant background reconstruction. To improve historical retrieval from the corrupted current-frame feature, we form the perturbed query using a learnable query projection *W*_*q*_, together with a zero-initialized residual query branch that produces the perturbation Δ*Q*. The attention mechanism retrieves information from history $X_{\mathrm{hist}}$ using the perturbed query:

(13) $${\hat{F}}_{\mathrm{rec}}^{\left(t\right)}=\text{MLP}\left(\text{Attention}\left(Q=W_{q}{\tilde{F}}^{\left(t\right)}+\Delta Q,K=X_{\mathrm{hist}},V=X_{\mathrm{hist}}\right)\right)$$

This reconstructed feature ${\hat{F}}_{\mathrm{rec}}^{\left(t\right)}$ is then supervised by a reconstruction loss $L_{\mathrm{rec}}$ against the original clean features, explicitly guiding the model to utilize historical context for target calibration.

#### 2.4.3 Multi-task optimization strategy

The DART-refined pseudo-labels and their associated uncertainty estimates provide the supervision for the optimization described below. To fully exploit the massive semi-supervised augmented data while mitigating inherent pseudo-label noise, we employ a multi-task objective imposing dual constraints on semantic alignment and temporal consistency. The primary objective is an uncertainty-weighted detection loss. The detection head takes the temporally enhanced feature $F_{\mathrm{out}}^{\left(t\right)}$ from the main stream as input. For retained pseudo-labeled samples, the frame-level uncertainty U(t) derived in Eq. (1) is used to down-weight less reliable supervision:

(14) $$L_{\det}=\underset{k}{\sum }{\left(1-U\left(t\right)\right)}^{\beta }\cdot L_{\mathrm{Y OLO}}\left(p_{k}^{\left(t\right)},{\hat{x}}_{k}^{\left(t\right)}\right)$$

Here, $p_{k}^{\left(t\right)}$ and ${\hat{x}}_{k}^{\left(t\right)}$ represent the prediction and the pseudo-label for the *k*-th object, respectively. The term ${\left(1-U\left(t\right)\right)}^{\beta }$ provides uncertainty-aware weighting for the retained pseudo-labeled samples, with *β* controlling the modulation strength. This weighting suppresses gradients from less reliable pseudo-labels.

Parallel to the detection task, the auxiliary stream performs masked feature reconstruction. The goal is to encourage the attention mechanism to capture temporal correspondences by recovering masked object-region features. The reconstruction objective encourages the reconstructed feature ${\hat{F}}_{\mathrm{rec}}^{\left(t\right)}$ to match the uncorrupted current-frame feature $F_{\mathrm{cur}}^{\left(t\right)}$ within the masked regions. The Mean Squared Error (MSE) is adopted as the consistency constraint:

(15) $$L_{\mathrm{rec}}=\frac{{\left\|\left({\hat{F}}_{\mathrm{rec}}^{\left(t\right)}-\text{sg}\left[F_{\mathrm{cur}}^{\left(t\right)}\right]\right)⊙M\right\|}_{2}^{2}}{\sum M+\epsilon }$$

where sg[⋅] denotes the stop-gradient operation, ensuring that $F_{\mathrm{cur}}^{\left(t\right)}$ serves as a fixed reconstruction target, and *ɛ* is a small positive constant for numerical stability. This constraint encourages the network to recover masked object-region features from temporally corresponding historical cues.

The total objective is defined as

(16) $$L_{\mathrm{total}}=L_{\det}+\lambda_{\mathrm{rec}}L_{\mathrm{rec}}$$

where *λ*_*rec*_ controls the contribution of the auxiliary reconstruction objective.

### 2.5 Missing-seedling gap inference via dual-scale kinematics

Although MTI-Net enhances visible-seedling perception, the resulting detections remain discrete spatial observations affected by localization noise and field-level geometric variation. To infer missing-seedling gaps from these detections, we propose the Dual-Scale Inertial Metric (DS-IM), which combines macro-scale row-periodicity reconstruction with micro-scale trajectory and growth-space verification.

#### 2.5.1 Macro-scale: periodic grid reconstruction

We first reconstruct the field layout by exploiting the periodicity of mechanical sowing. An adaptive dynamic window algorithm is deployed to partition inter-row boundaries based on local spatial density profiles, effectively aggregating discrete seedling detections into coherent, longitudinal row trajectories. To rectify row-level discontinuities (e.g., entire row absence), we introduce a global periodicity constraint. By statistically inferring the baseline row spacing Δ*Y*_*base*_, we perform a periodic verification on the spatial span between adjacent dynamic windows:

(17) $$N_{\text{miss}}=\max\left(0,\text{Round}\left(\frac{D_{\text{gap}}}{\Delta Y_{\text{base}}}\right)-1\right)$$

where *D*_gap_ denotes the spatial distance between the centroids of two adjacent detected rows, and *N*_miss_ represents the estimated number of missing rows. When *N*_miss_ ≥ 1, the algorithm identifies a candidate row-level absence and reconstructs the corresponding virtual row indices according to the statistically estimated row periodicity. This procedure reduces false gap candidates caused by incomplete visible-seedling detections.

#### 2.5.2 Micro-scale: trajectory-guided verification

Transitioning to a localized perspective, we fit a row-wise inertial trajectory to represent the expected mechanical sowing path and suppress local spatial deviations [53]. Virtual plant hypotheses *b*_virt_ are then generated within candidate gap regions. Their sizes are adaptively determined by combining the 95th percentile of the global seedling-height distribution with an upper-bound height statistic from the corresponding row. The resulting scale is used to construct a square virtual bounding box for each candidate.

To distinguish feasible gaps from visually empty but spatially constrained regions, a collision-based spatial verification is performed:

(18) $$\text{Valid}\left(b_{\text{virt}}\right)=I\left[\left(b_{\text{virt}}⊕\delta_{r}\right)\cap b_{k}=\emptyset ,\;\forall b_{k}\in B_{\text{obs}}\cup B_{\text{gen}}\right],$$

where I[⋅] denotes the indicator function, and ⊕ *δ*_*r*_ denotes spatial expansion of the virtual box by the scale-adaptive growth-space margin *δ*_*r*_. $B_{\text{obs}}$ is the set of observed seedlings, whereas $B_{\text{gen}}$ contains the previously accepted virtual hypotheses. The condition Valid(*b*_virt_) = 1 indicates that the candidate location provides sufficient non-overlapping space for a missing-seedling hypothesis.

## 3 Experiments and results

### 3.1 Experimental settings

#### 3.1.1 Implementation details

Experiments were conducted on an NVIDIA RTX 4090 (24 GB) GPU using Ubuntu 20.04, PyTorch 2.0, and CUDA 11.8. All baseline models were evaluated under a unified configuration to ensure fair comparison. These baselines include the YOLO series (YOLOv8 to YOLO13) [[54], [55], [56], [57], [58], [59]], YOLO-Master [60], the Transformer-based RT-DETR [61], and SSP-MambaNet [62]. Specific training hyperparameters were configured as follows: the AdamW optimizer was employed with an initial learning rate of 1 × 10^−3^, first- and second-moment coefficients of 0.937 and 0.999, respectively, and a weight decay coefficient of 5 × 10^−4^. The learning rate scheduling strategy utilized cosine annealing, with a total of 150 training epochs.

Unless otherwise specified, all input images were resized to 640 × 640 pixels, and the batch size was set to 8. For the proposed framework, the object-aware masking operation in the MTI-Net auxiliary branch was stochastically activated with a probability of 50% during training. Unless otherwise specified, the main comparative, cross-architecture, ablation, and label-efficiency experiments were repeated with five random seeds (42, 43, 44, 45, and 46), and the results are reported as mean ± standard deviation. For the external semi-supervised comparison, we implemented Efficient Teacher [63] and the SMT-inspired temporal mean-teacher baseline [22]. The supervised, Efficient Teacher, SMT-inspired, and DART-only settings used identical YOLO12-S backbones, labeled sparse-anchor partitions, training schedules, and random seeds. The three semi-supervised settings additionally shared the same unlabeled-frame pool. The corresponding seed-paired effect estimates are reported in Table S2. The public repository provides the model configuration files, training settings, and links to the source implementations of the evaluated detectors. Edge-device inference was additionally evaluated on an NVIDIA Jetson Nano using TensorRT FP16. Each configuration was profiled over 1,000 inference runs, with the first 50 runs excluded as warm-up.

#### 3.1.2 Evaluation metrics

Detection performance is evaluated using mAP@0.50, mAP@[0.5:0.95], and Recall. We treat mAP@[0.5:0.95] as the primary accuracy metric because it evaluates localization quality across multiple IoU thresholds. Computational efficiency is assessed using parameter count, GFLOPs, batch-1 CUDA latency, peak incremental GPU memory, and edge-device latency and FPS at an input resolution of 640 × 640. DS-IM is evaluated separately using gap-level Precision, Recall, and F1-score under one-to-one matching, together with frame-level gap-presence metrics and gap-count MAE and RMSE.

### 3.2 Comparison with representative detectors

As shown in Table 1, the proposed method achieves the best overall detection performance among the evaluated lightweight detectors. With YOLO12-S, the full framework reaches 92.92 ± 0.17% mAP@0.50, 69.33 ± 0.22% mAP@[0.5:0.95], and 86.59 ± 0.41% Recall, improving the baseline by +1.31, +1.88, and +2.80 pp, respectively. The proposed model requires 23.5 GFLOPs and an inference latency of 17.3 ms per image, compared with 21.5 GFLOPs and 13.2 ms for the YOLO12-S baseline. Its latency remains lower than those of SSP-MambaNet-S and RT-DETR-N, which require 24.1 and 26.5 ms per image, respectively. Under batch-1 CUDA inference, the peak incremental GPU memory increased from 771 MB for the YOLO12-S baseline to 807 MB for the proposed model, corresponding to an additional 36 MB. Detailed cross-architecture memory results are provided in Fig. S4. Overall, the accuracy improvements are accompanied by a moderate increase in runtime and computational complexity. On the Jetson Nano, the YOLO12-S baseline achieves 114.55 ms inference latency and 8.77 FPS, whereas Ours requires 423.47 ms and achieves 2.36 FPS under TensorRT FP16, indicating that the current implementation is more suitable for sampled or low-rate field inspection than for high-frame-rate processing.

**Table 1:** Comparison with representative lightweight detectors. Accuracy metrics are reported in %, Params in millions, GFLOPs at 640 × 640, and latency in ms/img. All reported differences are computed from unrounded values.

Method	mAP@0.50	mAP@[0.5:0.95]	Recall	Params	GFLOPs	Lat.
*Transformer*						
RT-DETR-N	91.07 ± 0.61	67.75 ± 0.51	85.11 ± 1.07	19.6	67.4	26.5

*Existing Variants*						
SSP-MambaNet-S	91.67 ± 0.27	59.61 ± 0.31	85.36 ± 0.53	9.8	58.2	24.1

*YOLO Series*						
YOLOv8-S	91.86 ± 0.43	68.07 ± 0.66	85.32 ± 0.90	11.1	28.6	6.3
YOLOv9-S	92.27 ± 0.79	68.10 ± 1.02	85.29 ± 1.36	7.2	27.4	16.3
YOLOv10-S	91.46 ± 0.34	67.75 ± 0.31	83.08 ± 0.99	8.0	24.8	8.5
YOLO11-S	92.12 ± 0.20	68.05 ± 0.32	84.62 ± 0.66	9.4	21.5	8.5
YOLO12-S (Base)	91.61 ± 0.53	67.45 ± 0.75	83.78 ± 0.43	9.3	21.5	13.2
YOLO13-S	89.37 ± 0.60	61.48 ± 1.17	82.26 ± 0.50	9.0	21.0	18.4
YOLO-Master-S	90.84 ± 0.48	64.59 ± 0.68	82.35 ± 0.86	9.6	29.2	17.8

**Ours (YOLO12-S)**	**92.92 ± 0.17 (**+1.31)	**69.33 ± 0.22 (**+1.88)	**86.59 ± 0.41 (**+2.80)	12.1	23.5	17.3

Qualitative comparisons in challenging field scenarios (Fig. 3) show that the proposed method identifies more fragmented or low-response seedlings than representative baselines under low-contrast and dense conditions, consistent with the observed improvement in Recall.

**Fig. 3:**
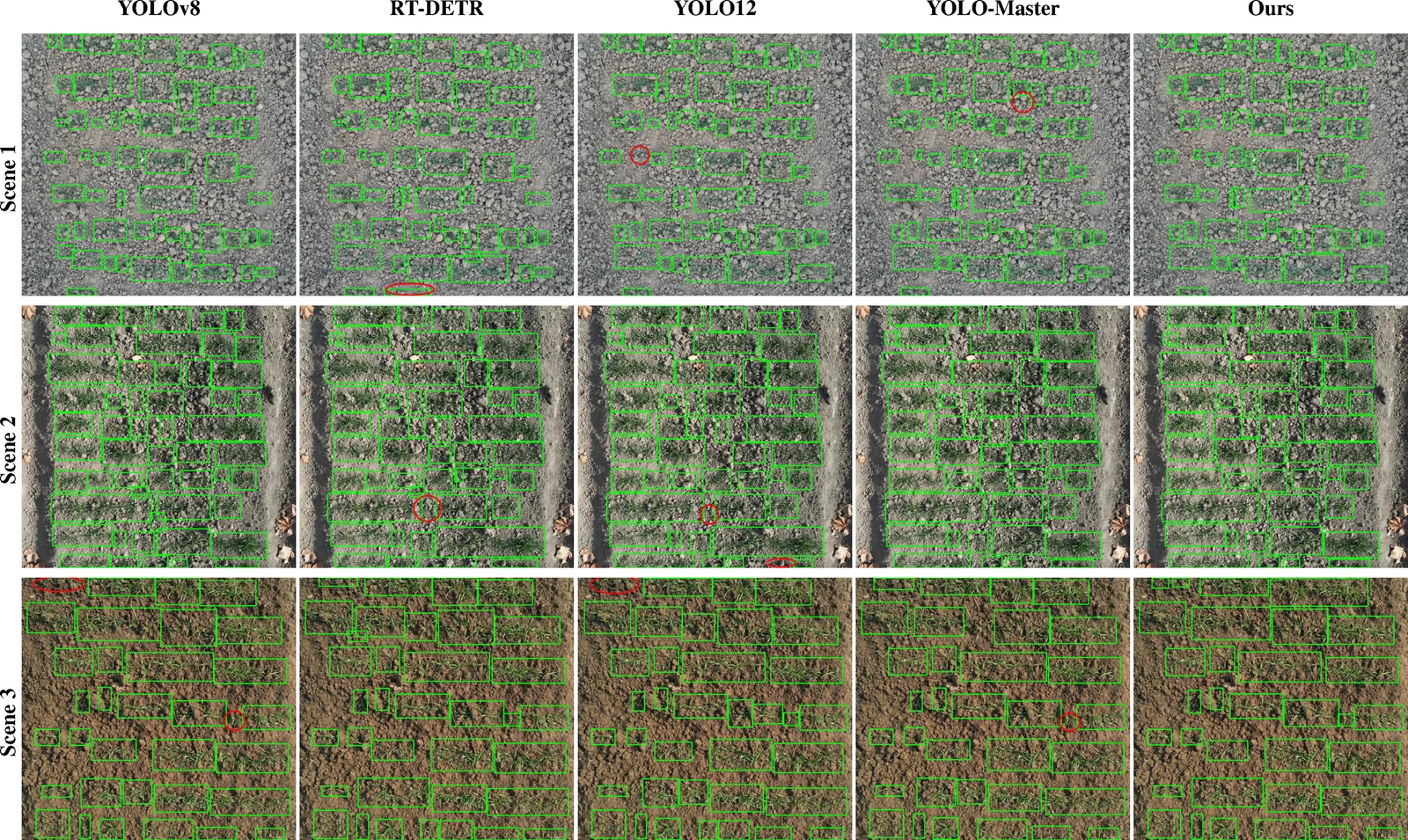
Qualitative comparison in challenging scenarios. Compared with representative baseline models, our method identifies more weak or fragmented seedling targets under dense sowing and low-contrast backgrounds.

### 3.3 Cross-architecture transferability

To evaluate cross-architecture adaptability, we embedded the proposed modules into representative real-time detection architectures, including RT-DETR, YOLOv8–YOLO13, and YOLO-Master. As shown in Table 2, positive mean changes in mAP@[0.5:0.95] are observed across all evaluated detector backbones. For YOLO-Master-S, the framework yields mean gains of +4.50 percentage points in mAP@[0.5:0.95] and +3.94 percentage points in Recall. On the primary YOLO12-S backbone, the corresponding gains are +1.88 and + 2.80 percentage points. RT-DETR-N shows a smaller mean change of +0.31 percentage points in mAP@[0.5:0.95], together with a +1.97-percentage-point gain in Recall. The consistent direction of the mean changes across Transformer-based and YOLO-family detectors supports the cross-architecture adaptability of the proposed framework. Complete seed-paired effect estimates are provided in Table S2.

**Table 2:** Transferability across RT-DETR and YOLO variants. YOLO-M denotes YOLO-Master. All reported differences are computed from unrounded values.

Model	Setup	Metrics (%)		
		mAP@0.50	mAP@[0.5:0.95]	Recall
RT-DETR	Base	91.07 ± 0.61	67.75 ± 0.51	85.11 ± 1.07
	**Ours**	**91.72 ± 0.26 (**+0.66)	**68.06 ± 0.39 (**+0.31)	**87.07 ± 0.50 (**+1.97)
YOLOv8	Base	91.86 ± 0.43	68.07 ± 0.66	85.32 ± 0.90
	**Ours**	**92.54 ± 0.33 (**+0.67)	**69.22 ± 0.18 (**+1.14)	**86.48 ± 0.40 (**+1.16)
YOLOv9	Base	92.27 ± 0.79	68.10 ± 1.02	85.29 ± 1.36
	**Ours**	**92.83 ± 0.21 (**+0.56)	**69.48 ± 0.29 (**+1.38)	**86.76 ± 0.26 (**+1.47)
YOLOv10	Base	91.46 ± 0.34	67.75 ± 0.31	83.08 ± 0.99
	**Ours**	**92.39 ± 0.08 (**+0.93)	**69.09 ± 0.03 (**+1.34)	**85.96 ± 0.33 (**+2.88)
YOLO11	Base	92.12 ± 0.20	68.05 ± 0.32	84.62 ± 0.66
	**Ours**	**92.94 ± 0.15 (**+0.83)	**69.47 ± 0.24 (**+1.42)	**87.09 ± 0.38 (**+2.47)
YOLO12	Base	91.61 ± 0.53	67.45 ± 0.75	83.78 ± 0.43
	**Ours**	**92.92 ± 0.17 (**+1.31)	**69.33 ± 0.22 (**+1.88)	**86.59 ± 0.41 (**+2.80)
YOLO13	Base	89.37 ± 0.60	61.48 ± 1.17	82.26 ± 0.50
	**Ours**	**92.08 ± 0.19 (**+2.71)	**66.49 ± 0.69 (**+5.01)	**85.54 ± 0.46 (**+3.28)
YOLO-M	Base	90.84 ± 0.48	64.59 ± 0.68	82.35 ± 0.86
	**Ours**	**92.78 ± 0.25 (**+1.94)	**69.09 ± 0.25 (**+4.50)	**86.30 ± 0.48 (**+3.94)

### 3.4 Ablation studies

We conducted ablation experiments on YOLO12-S to evaluate the individual and combined contributions of DART and MTI-Net. Additional controls were introduced to examine whether the effectiveness of MTI-Net arises solely from temporal aggregation or a generic reconstruction task. All results are reported as mean ± standard deviation over five random seeds.

As shown in Table 3, DART and MTI-Net yield mean gains of 0.86 and 1.52 percentage points in mAP@[0.5:0.95] over YOLO12-S, respectively. The combined configuration attains the largest mean values among the component settings, reaching 69.33 ± 0.22% mAP@[0.5:0.95] and 86.59 ± 0.41% Recall.

**Table 3:** Ablation study of the proposed components on YOLO12-S. All reported differences are computed from unrounded values.

Setting	mAP@0.50 (%)	mAP@[0.5:0.95] (%)	Recall (%)
*Component ablation*			
YOLO12-S	91.61 ± 0.53	67.45 ± 0.75	83.78 ± 0.43
+ DART	92.45 ± 0.32 **(**+0.84)	68.31 ± 0.40 **(**+0.86)	85.29 ± 0.81 **(**+1.50)
+ MTI-Net	92.71 ± 0.36 **(**+1.10)	68.97 ± 0.43 **(**+1.52)	86.04 ± 0.39 **(**+2.25)
**+ DART + MTI-Net**	**92.92 ± 0.17 (**+1.31)	**69.33 ± 0.22 (**+1.88)	**86.59 ± 0.41 (**+2.80)

*MTI-Net internal controls without DART*			
Temporal aggregation	92.65 ± 0.35	68.56 ± 0.56	85.77 ± 0.26
Random-box reconstruction	92.31 ± 0.28	68.27 ± 0.16	85.90 ± 0.55

The matched controls further separate the roles of temporal aggregation and object-aware reconstruction. Temporal aggregation reaches 68.56 ± 0.56% mAP@[0.5:0.95], while the random-box reconstruction control reaches 68.27 ± 0.16%. The complete MTI-Net configuration reaches 68.97 ± 0.43%. Relative to random-box reconstruction, object-aware masking provides a paired mean gain of 0.70 percentage points in mAP@[0.5:0.95]. Taken together, these results show that MTI-Net builds on temporal context aggregation while its detection-oriented object-region reconstruction provides additional benefit. Complete paired estimates, including the DART stage-wise comparisons, are reported in Table S2, while the stage-wise performance of DART and DS-IM is summarized in Table S6.

### 3.5 Semi-supervised learning efficiency

#### 3.5.1 Labeling efficiency quantitative analysis

To quantify labeling efficiency, we evaluated the proposed framework at 10%, 25%, 50%, and 100% of the complete sparse-anchor annotation pool. The 100% setting denotes all manually annotated keyframes used for training rather than dense frame-by-frame annotation. The four settings contain 4,837, 11,886, 23,482, and 46,676 annotated bounding boxes, respectively, with estimated active annotation costs of 16.7, 40.8, 80.6, and 160.1 human-hours under the assumptions summarized in Table S1.

As illustrated in Fig. 4a and b, the proposed framework yields higher mean detection performance across all sparse-anchor annotation ratios. Under the most label-limited 10% setting, mAP@[0.5:0.95] increases from 48.31 ± 0.97% to 59.83 ± 0.60% (+11.51 pp), while Recall increases from 75.23 ± 1.06% to 82.98 ± 0.35% (+7.75 pp). With only 50% of the sparse-anchor annotations, the framework achieves 66.78 ± 0.42% mAP@[0.5:0.95], approaching the 100% sparse-anchor baseline of 67.45 ± 0.75%, and reaches 86.12 ± 0.54% Recall, surpassing the corresponding baseline of 83.78 ± 0.43%. Under the full sparse-anchor setting, the complete framework further improves mAP@[0.5:0.95] to 69.33 ± 0.22%. These results indicate that the proposed framework can reduce sparse-anchor labeling effort while maintaining reliable visible-seedling perception, especially in label-limited training scenarios.

**Fig. 4:**
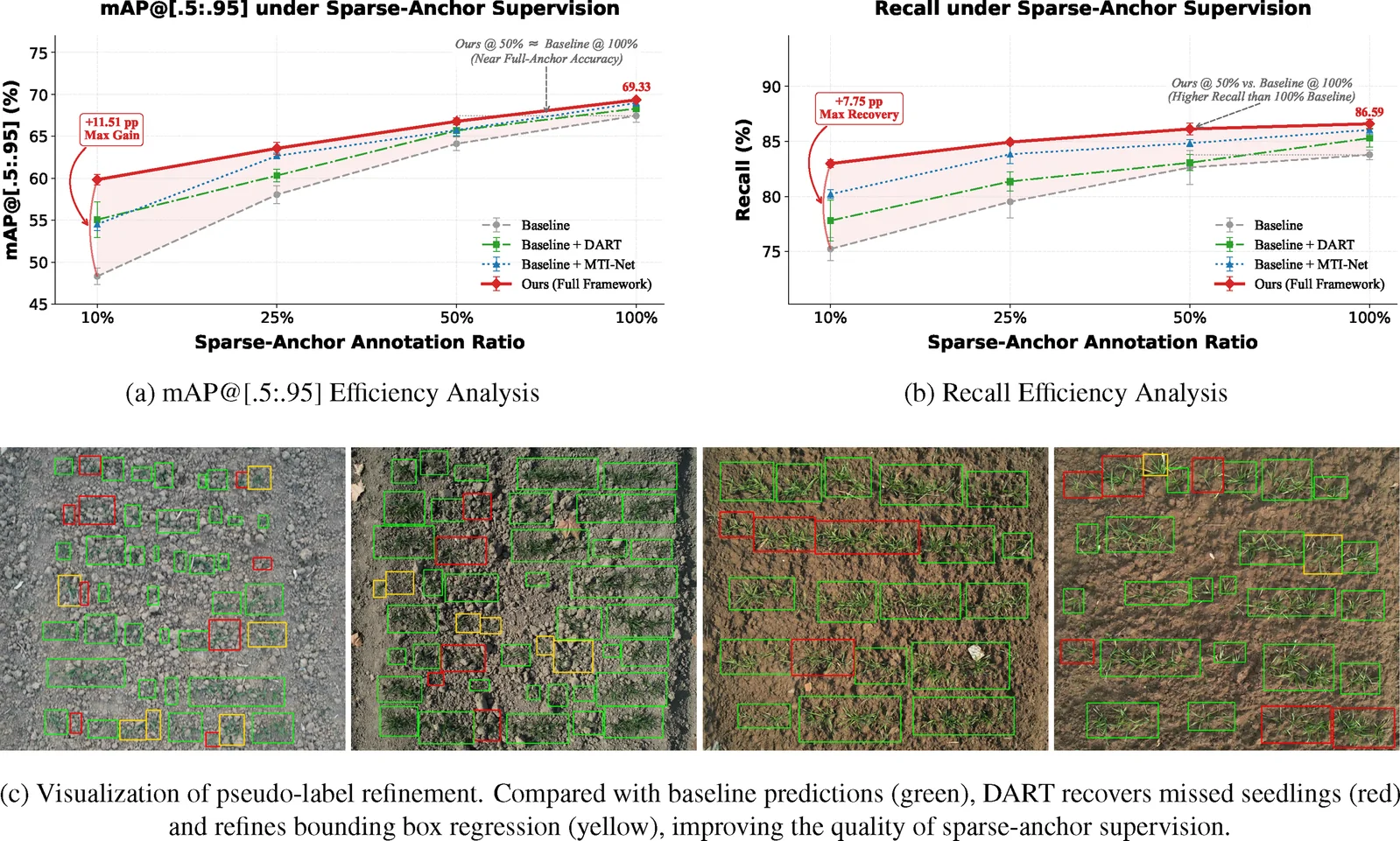
Label-efficiency evaluation under different sparse-anchor annotation ratios. (a) mAP@[0.5:0.95] under 10%, 25%, 50%, and 100% sparse-anchor annotations. (b) Recall under different sparse-anchor annotation ratios. (c) Qualitative visualization of pseudo-label refinement.

To provide complementary semi-supervised references, we compared DART with Efficient Teacher [63] and the SMT-inspired temporal mean-teacher baseline [22] under the matched protocol described in Section 3.1.1. DART-only achieves higher mean mAP@[0.5:0.95] than Efficient Teacher at the 10%, 25%, and 50% settings, with gains of 7.36, 1.92, and 2.33 percentage points, respectively. At 25%, DART-only reaches 60.32 ± 0.74% mAP@[0.5:0.95], compared with 58.40 ± 1.72% for Efficient Teacher and 58.47 ± 1.62% for the SMT-inspired baseline. Complete results and paired estimates are reported in Tables S2 and S5.

#### 3.5.2 Validation of temporal uncertainty and shunting

We evaluated the temporal uncertainty assumption using the low-disagreement trajectories retained after shunting over propagation distances of 1–60 frames. For the normalized squared center residual, the bin-level Pearson correlations *r*_bin_ were 0.873 and 0.860 for the forward and backward directions, while the corresponding count-weighted linear fits yielded $R_{w}^{2}$ values of 0.657 and 0.625. For 1 − IoU, the corresponding *r*_bin_ values were 0.951 and 0.866, with $R_{w}^{2}$ values of 0.891 and 0.731. These results provide empirical support for an approximately increasing uncertainty trend with temporal distance within the retained short-interval regime.

We further used manually annotated frames withheld from trajectory initialization to evaluate bidirectional disagreement against actual tracking error. Across the 10%, 25%, and 50% sparse-anchor settings, the disagreement measure achieved Spearman correlations of 0.388–0.426 with hidden-annotation error and AUROC values of 0.757–0.766 for identifying predictions with IoU < 0.5. The retained predictions achieved mIoU values of 0.727–0.771, compared with 0.263–0.281 for the rejected predictions, while only 1.8%–2.4% of predictions were rejected. Detailed temporal trends and the retained-versus-rejected comparison are provided in Fig. S1.

#### 3.5.3 Visual analysis of sample refinement

Fig. 4c illustrates the pseudo-label refinement produced by DART. Green boxes denote high-confidence baseline predictions, red boxes indicate seedlings recovered through bidirectional temporal consistency, and yellow boxes show localization refinement. Because only low-disagreement predictions enter the refinement stage, these additional samples primarily expand supervision for missed or poorly localized seedlings without requiring dense frame-level annotation.

### 3.6 Spatiotemporal mechanism insight

Fig. 5 visualizes the cross-frame attention, fusion gating, and object-region reconstruction responses of MTI-Net. Historical features receive greater attention near weakened current-frame seedling responses, while the fusion gate assigns stronger activations to regions requiring temporal compensation. In the reconstruction examples, the corrupted target features are partially restored using temporally corresponding historical cues. These qualitative observations complement the matched controls by illustrating how the complete MTI-Net design combines historical feature retrieval with detection-oriented object-region reconstruction.

**Fig. 5:**
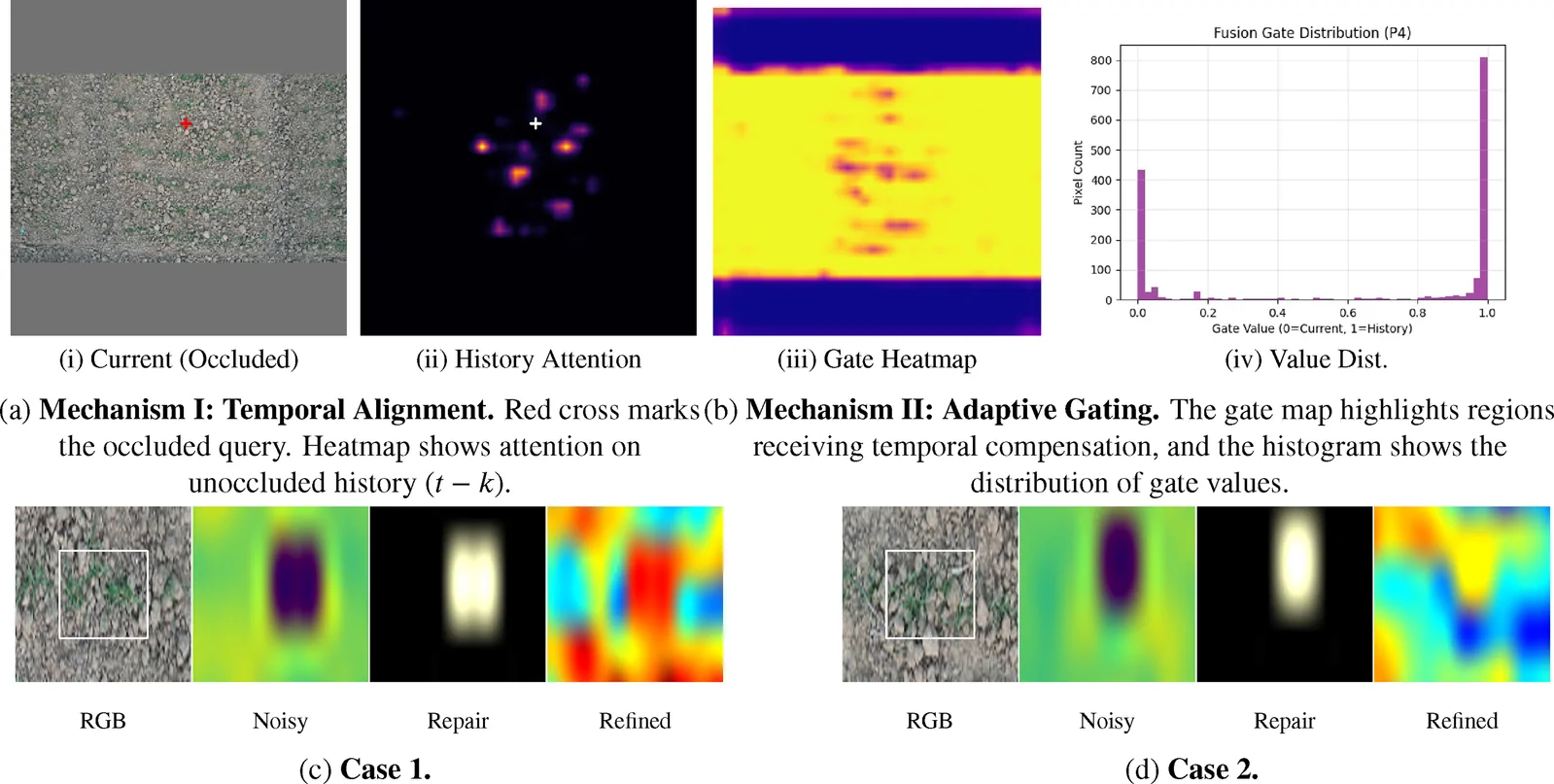
**MTI-Net feature visualization.** (a) Cross-frame attention around a weakened current-frame seedling response. (b) Fusion gate responses and their value distribution. (c–d) Feature responses before and after object-aware reconstruction.

### 3.7 Robustness and sensitivity analysis

#### 3.7.1 Hyperparameter sensitivity

We analyzed the sensitivity of MTI-Net to the frame sampling interval Δ*t* and history length *K*, as shown in Fig. 6a. Performance was lower at Δ*t* = 1, improved at Δ*t* = 4, and declined when the interval increased to 7, indicating reduced effectiveness when adjacent frames are either highly redundant or temporally distant. Increasing *K* from 1 to 4 improved both mAP and Recall, whereas extending it to 7 produced only a marginal mAP change and a slight Recall reduction. We therefore adopted Δ*t* = 4 and *K* = 4 as a balance between detection performance and computational cost.

**Fig. 6:**
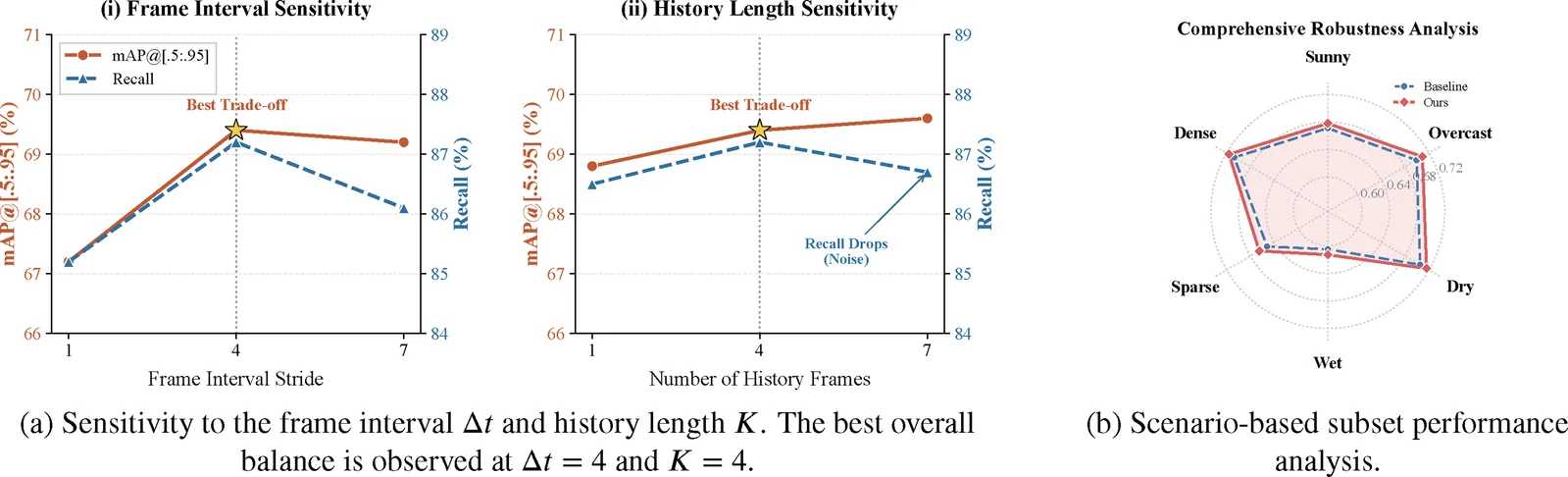
Robustness and sensitivity analysis of the proposed framework. The results show the effects of temporal hyperparameters and environmental conditions on detection performance.

#### 3.7.2 Cross-field applicability

To evaluate cross-field adaptability, we conducted bidirectional leave-one-field-out experiments using YOLO12-S over five random seeds. When trained on Field 2 and evaluated on Field 1, mean mAP@[0.5:0.95] increased from 56.03 ± 1.75% to 58.28 ± 1.40%, while Recall increased from 75.77 ± 1.42% to 79.41 ± 0.21%. In the reverse direction, the corresponding values increased from 46.38 ± 1.10% to 48.87 ± 1.09% and from 74.07 ± 2.20% to 77.26 ± 0.62%, respectively. Positive mean changes across both transfer directions support within-region cross-field adaptability. Complete results and seed-paired estimates are provided in Fig. S3.

#### 3.7.3 Scenario-based performance stratification

To evaluate performance variations under different field conditions, we analyzed six subsets stratified by illumination, soil appearance, and seedling density. As shown in Fig. 6b, the proposed method maintains positive gains over the baseline across all evaluated subsets. For example, mAP@[0.5:0.95] increases from 60.5% to 61.3% on wet soil and from 65.2% to 66.5% in sparse-seedling scenes, indicating that the improvements are not confined to a single environmental condition.

### 3.8 Agronomic evaluation of missing-seedling gap inference

#### 3.8.1 Quantitative evaluation of gap inference

DS-IM was evaluated using a one-to-one Hungarian matching protocol. A predicted gap was considered correctly localized when its center fell within an unmatched ground-truth gap region, while unmatched predictions and annotations were treated as false positives and false negatives, respectively. Detailed matching results under alternative criteria are provided in Table S3. Under the primary criterion, DS-IM achieved a Precision of 86.73%, a Recall of 82.43%, and an F1-score of 84.53%.

For frame-level gap-presence evaluation, the corresponding Precision, Recall, and F1-score were 91.43%, 93.20%, and 92.31%, respectively. The gap-count MAE and RMSE were 0.229 and 0.556 gaps per frame, and the predicted count differed from the annotated count by no more than one gap in 96.86% of the evaluated frames. Complete frame-level metrics are reported in Table S4, and representative gap-inference results are presented in Fig. 7a.

**Fig. 7:**
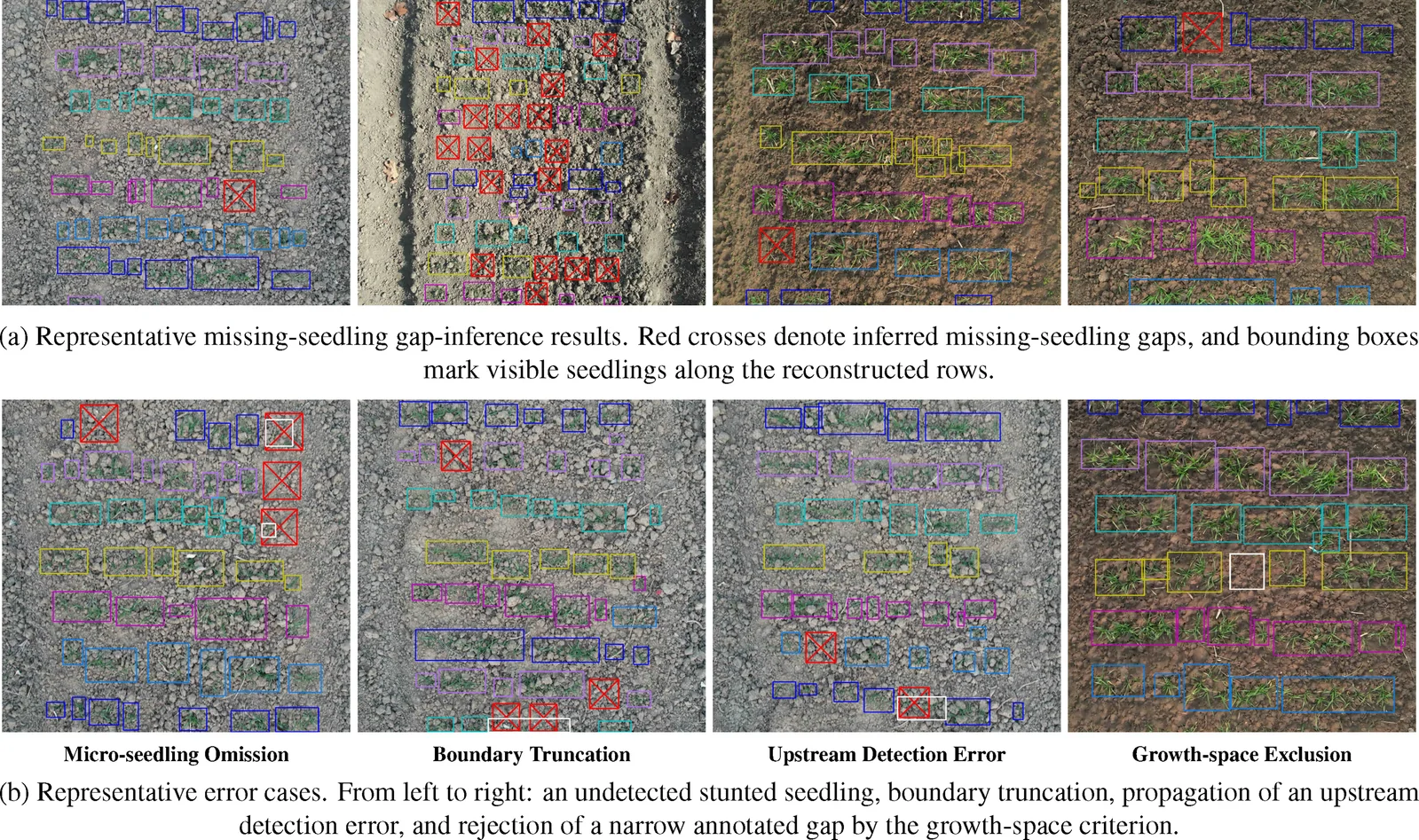
Qualitative evaluation of the proposed DS-IM module. (a) Representative missing-seedling gap-inference results. (b) Typical error cases under challenging conditions.

We further examined the sensitivity of DS-IM to its adjustable geometric and scale-related settings. The row-splitting factor and row-extent percentile produced comparatively limited variations within the evaluated ranges, whereas the global height percentile and growth-space safety margin had stronger effects on the gap-acceptance behavior. Smaller effective spatial margins tended to admit additional gap candidates, while larger margins produced more conservative predictions. The complete one-factor-at-a-time sensitivity and interaction analyses are provided in Fig. S2.

To further examine cross-crop applicability, DS-IM was evaluated on an external maize seedling dataset from Seedling-DETR [64], with crop-specific geometric calibration using a fixed 10% subset. On 817 annotated images, DS-IM achieved 89.38% Precision, 75.08% Recall, 81.61% F1-score, and a count MAE of 1.721 gaps/frame under the same center-in-GT matching protocol.

#### 3.8.2 Error analysis and limitations

The representative cases in Fig. 7b illustrate three sources of false-positive gap predictions: missed small seedlings, boundary truncation, and persistent upstream detection errors. In Fig. 7b (Micro-seedling Omission), a stunted or micro-scale seedling is missed by the upstream detector, causing its location to be interpreted as empty by DS-IM. In Fig. 7b (Boundary Truncation), incomplete observations near the image or field boundary create an artificial interruption in the reconstructed spatial pattern. In Fig. 7b (Upstream Detection Error), persistent omissions or false detections propagate from visible-seedling perception to the downstream gap-inference stage. Temporal consistency can mitigate isolated frame-level fluctuations, but it cannot fully compensate for persistent upstream errors. From a downstream decision perspective, these false positives may overestimate local reseeding demand.

Representative false-negative cases are associated with the adaptive growth-space criterion, as illustrated in Fig. 7b (Growth-space Exclusion). Because the virtual seedling scale is estimated from the current crop population, a narrow annotated gap may fail the non-overlap requirement when surrounding plants exhibit relatively large spatial occupancy and is consequently rejected. This criterion reflects a conservative balance between gap sensitivity and spatial feasibility: it reduces the likelihood of treating every local discontinuity as an admissible gap location, but may also lower sensitivity to narrow gaps and thereby underestimate the extent of a local stand deficit. For field deployment, consistent imaging geometry and crop-specific spatial calibration should be maintained. A feasible on-site validation strategy is to georeference the inferred gaps and compare them with independent field observations from matched row segments, jointly assessing localization and counting consistency together with processing efficiency.

## 4 Conclusion

This study presents a detection-driven framework integrating DART, MTI-Net, and DS-IM for label-efficient visible-seedling perception and missing-seedling gap inference in UAV wheat videos. When integrated with YOLO12-S, the framework achieves 92.92 ± 0.17% mAP@0.50, 69.33 ± 0.22% mAP@[0.5:0.95], and 86.59 ± 0.41% Recall. DS-IM further achieves an F1-score of 84.53% and a frame-level count MAE of 0.229 gaps/frame. The results show positive mean improvements across detector backbones, sparse-anchor ratios, and the two evaluated fields.

The current evaluation covers early-stage nadir-view videos from two fields within one region. Future work will extend validation to additional regions, growth stages, flight configurations, and field-level reseeding outcomes.

## Authors’ contributions

Y. Nie led conceptualization, methodology, formal analysis, and original drafting. H. Ma provided overall supervision, research design, and critical review. X. Wang and P. Zhang handled data curation, validation, and resources. Y. Wu conducted software and visualization. X. Li and L. Gu assisted with methodology and manuscript revision. All authors approved the final manuscript.

## Data availability

Our data and code are available at: https://github.com/ezioyao/MTI-Net.

## Funding

This study was funded by the Support Program for Excellent Young Talents in Colleges and Universities of Anhui Province (grant number rk482201) and the Open Research Fund of Henan Zhongke Science and Technology Achievement Transfer and Transformation Center (grant number 2024134).

## Declaration of competing interest

The authors declare that they have no known competing financial interests or personal relationships that could have appeared to influence the work reported in this paper.

## References

[1] McDonald G.K., Minkey D., Desbiolles J., Clarke G., Allen R., Noack S., Schmitt S., Amougis A. (2024). Responses to precision planting in canola and grain legume crops. Field Crops Res..

[2] Jin X., Liu S., Baret F., Hemerlé M., Comar A. (2017). Estimates of plant density of wheat crops at emergence from very low altitude uav imagery. Rem. Sens. Environ..

[3] Wilke N., Siegmann B., Postma J.A., Muller O., Krieger V., Pude R., Rascher U. (2021). Assessment of plant density for barley and wheat using uav multispectral imagery for high-throughput field phenotyping. Comput. Electron. Agric..

[4] Feng A., Zhou J., Vories E., Sudduth K.A. (2020). Evaluation of cotton emergence using uav-based imagery and deep learning. Comput. Electron. Agric..

[5] Zhang P., Sun X., Zhang D., Yang Y., Wang Z. (2023). Lightweight deep learning models for high-precision rice seedling segmentation from uav-based multispectral images. Plant Phenomics.

[6] Osco L.P., Marcato Junior J., Marques Ramos A.P., de Castro Jorge L.A., Fatholahi S.N., de Andrade Silva J., Matsubara E.T., Pistori H., Gonçalves W.N., Li J. (2021). A review on deep learning in uav remote sensing. Int. J. Appl. Earth Obs. Geoinf..

[7] Wu Y., Yuan S., Tang L. (2025). Plant recognition of maize seedling stage in uav remote sensing images based on h-rt-detr. Plant Methods.

[8] Ubbens J., Stavness I., Pound M.P., Guo W. (2025). Deep learning in plant phenotyping: the first ten years. Plant Phenomics.

[9] Yuan J., Li X., Zhou M., Zheng H., Liu Z., Liu Y., Wen M., Cheng T., Cao W., Zhu Y., Yao X. (2024). Rapidly count crop seedling emergence based on waveform method(wm) using drone imagery at the early stage. Comput. Electron. Agric..

[10] Bai Y., Yu J., Yang S., Ning J. (2024). An improved yolo algorithm for detecting flowers and fruits on strawberry seedlings. Biosyst. Eng..

[11] Cheng G., Yuan X., Yao X., Yan K., Zeng Q., Xie X., Han J. (2023). Towards large-scale small object detection: survey and benchmarks. IEEE Trans. Pattern Anal. Mach. Intell..

[12] Xie J., Pang Y., Nie J., Cao J., Han J. (2023). Latent feature pyramid network for object detection. IEEE Trans. Multimed..

[13] Deng C., Wang M., Liu L., Liu Y., Jiang Y. (2022). Extended feature pyramid network for small object detection. IEEE Trans. Multimed..

[14] Li J., Yu C., Wei W., Li J., Huang K., Hussian A., Liu X., Zhou Y. (2025). A multi-scale feature fusion network focusing on small objects in uav-view. Cogn. Comput..

[15] Lu Y., Young S. (2020). A survey of public datasets for computer vision tasks in precision agriculture. Comput. Electron. Agric..

[16] Song G., Du H., Zhang X., Bao F., Zhang Y. (2024). Small object detection in unmanned aerial vehicle images using multi-scale hybrid attention. Eng. Appl. Artif. Intell..

[17] Qian J., Tao C., Luo X., Gao Z., Wang T., Xiao F., Cao F., Zhang Z. (2026). Dronet: occlusion-mastering multi-object detection tailored for unmanned aerial vehicles. Displays.

[18] Jiang L., Yuan B., Du J., Chen B., Xie H., Tian J., Yuan Z. (2024). Mffsodnet: multiscale feature fusion small object detection network for uav aerial images. IEEE Trans. Instrum. Meas..

[19] Cob-Parro A.C., Losada-Gutiérrez C., Marrón-Romera M., Gardel-Vicente A., Bravo-Muñoz I. (2024). A new framework for deep learning video based human action recognition on the edge. Expert Syst. Appl..

[20] Zheng W., Zheng H., Sun Y., Jing Y. (2025). Intstr: an integrated spatio-temporal relation transformer for video object detection. Neurocomputing.

[21] K S., K S., D. S R., B M., V P., R P. (2025). 2025 3rd International Conference on Integrated Circuits and Communication Systems (ICICACS).

[22] Kumar A., Mitra S., Rawat Y.S. (2025). https://ojs.aaai.org/index.php/AAAI/article/view/32465.

[23] Moorthy S., S. K.S S., Arthanari S., Jeong J.H., Joo Y.H. (2025). Hybrid multi-attention transformer for robust video object detection. Eng. Appl. Artif. Intell..

[24] Tan Z., Chen C., Wen K., Cheng Q., Fu Z. (2024). Bridging spatiotemporal feature gap for video salient object detection. Knowl. Base Syst..

[25] Fu Z., Shuo Y., Cao P., Wei J., Wang H., Zhang G. (2025). End-to-end video object detection based on dynamic anchor box spatiotemporal decoder and hybrid matching. Neurocomputing.

[26] Zhu X., Wang Y., Dai J., Yuan L., Wei Y. (2017). 2017 IEEE International Conference on Computer Vision (ICCV).

[27] Zhao G., Pietikainen M. (2007). Dynamic texture recognition using local binary patterns with an application to facial expressions. IEEE Trans. Pattern Anal. Mach. Intell..

[28] Sieberth T., Wackrow R., Chandler J.H. (2016). Automatic detection of blurred images in uav image sets. ISPRS J. Photogrammetry Remote Sens..

[29] Zhou K., Li L., Zhou W., Wang Y., Feng H., Li H. (2025). Lanetca: enhancing video lane detection with temporal context aggregation. IEEE Trans. Circ. Syst. Video Technol..

[30] He K., Chen X., Xie S., Li Y., Dollár P., Girshick R. (2022). 2022 IEEE/CVF Conference on Computer Vision and Pattern Recognition (CVPR).

[31] Muhtar D., Zhang X., Xiao P., Li Z., Gu F. (2023). Cmid: a unified self-supervised learning framework for remote sensing image understanding. IEEE Trans. Geosci. Rem. Sens..

[32] Huang Z., Jin X., Lu C., Hou Q., Cheng M.-M., Fu D., Shen X., Feng J. (2024). Contrastive masked autoencoders are stronger vision learners. IEEE Trans. Pattern Anal. Mach. Intell..

[33] Peng J., Zhao H., Zhao K., Wang Z., Yao L. (2024). Dynamic background reconstruction via masked autoencoders for infrared small target detection. Eng. Appl. Artif. Intell..

[34] Tian Y., Jiang X., Adamowski J.F., Sun S. (2025). 2025 ASABE Annual International Meeting.

[35] Ma C., Li H., Jiang J., Aybar C., Yao J., Camps-Valls G. (2025). Dynamics of masked image modeling in hyperspectral image classification. IEEE Trans. Geosci. Rem. Sens..

[36] Tang N.C., Hsu C.-T., Su C.-W., Shih T.K., Liao H.-Y.M. (2011). Video inpainting on digitized vintage films via maintaining spatiotemporal continuity. IEEE Trans. Multimed..

[37] Vidović I., Cupec R., Hocenski Željko (2016). Crop row detection by global energy minimization. Pattern Recogn..

[38] Liu Y., He P., Cao Y., Zhu C., Ding S. (2024). Evaluation model of rice seedling production line seeding quality based on deep learning. Appl. Sci..

[39] Yang R., Chen M., Lu X., He Y., Li Y., Xu M., Li M., Huang W., Liu F. (2025). Integrating uav remote sensing and semi-supervised learning for early-stage maize seedling monitoring and geolocation. Plant Phenomics.

[40] Zhang X., Wang Q., Li H., Lu C., Shi R., Dun Z., Wang W., Shang Z. (2026). Multi-task geometric regression with agronomic priors for crop row and missing seedling detection in maize-soybean strip intercropping. Comput. Electron. Agric..

[41] Fu D., Cai Y., Liang Z., Tang Z., Yao Z., Gong H., Hu R., Li J., Qi L. (2026). Prsgnet: a robust framework for crop row detection in complex field scenarios. Comput. Electron. Agric..

[42] Najafian K., Ghanbari A., Sabet Kish M., Eramian M., Shirdel G.H., Stavness I., Jin L., Maleki F. (2023). Semi-self-supervised learning for semantic segmentation in images with dense patterns. Plant Phenomics.

[43] Qin Y., Chen J., Wang C., Pan C. (2025). Bisam-cd: Zero-shot remote sensing change detection via bidirectional temporal memory in sam2. IEEE Trans. Geosci. Rem. Sens..

[44] Liu X., Yin J., Liu J., Ding P., Liu J., Liu H. (2021). Trajectorycnn: a new spatio-temporal feature learning network for human motion prediction. IEEE Trans. Circ. Syst. Video Technol..

[45] Gomes H.M., Grzenda M., Mello R., Read J., Le Nguyen M.H., Bifet A. (Nov. 2022). A survey on semi-supervised learning for delayed partially labelled data streams. ACM Comput. Surv..

[46] Liu K., Jin S., Fu Z., Chen Z., Jiang R., Ye J. (2023). 2023 IEEE/CVF International Conference on Computer Vision (ICCV).

[47] Zhang R., Xu C., Zhu H., Xu F., Yang W., Zhang H., Xia G.-S. (2025). Minimizing sample redundancy for label-efficient object detection in aerial images. IEEE Trans. Geosci. Rem. Sens..

[48] Aziz Z., Urmonov O., Sajid S., Kim H. (2026). Improving object detection of intelligent vehicles through self-training with accurate labeling and class balancing. IEEE Trans. Intell. Veh..

[49] Zhang K., Peng J., Fu J., Liu D. (2024). Exploiting optical flow guidance for transformer-based video inpainting. IEEE Trans. Pattern Anal. Mach. Intell..

[50] Lu Z., Shuai B., Chen Y., Xu Z., Modolo D. (2024). 2024 IEEE/CVF Conference on Computer Vision and Pattern Recognition (CVPR).

[51] Cao J., Pang J., Weng X., Khirodkar R., Kitani K. (2023). 2023 IEEE/CVF Conference on Computer Vision and Pattern Recognition (CVPR).

[52] Schiappa M.C., Rawat Y.S., Shah M. (Jul. 2023). Self-supervised learning for videos: a survey. ACM Comput. Surv..

[53] Zhang S., Liu Y., Xiong K., Tian Y., Du Y., Zhu Z., Du M., Zhai Z. (2024). A review of vision-based crop row detection method: focusing on field ground autonomous navigation operations. Comput. Electron. Agric..

[54] Varghese R., M S. (2024). 2024 International Conference on Advances in Data Engineering and Intelligent Computing Systems (ADICS).

[55] Wang C.-Y., Yeh I.-H., Liao H.-Y.M. (2024). Yolov9: learning what you want to learn using programmable gradient information. https://arxiv.org/abs/2402.13616.

[56] Wang A., Chen H., Liu L., Chen K., Lin Z., Han J., Ding G. (2024). Yolov10: Real-time end-to-end object detection. https://arxiv.org/abs/2405.14458.

[57] Khanam R., Hussain M. (2024). Yolov11: an overview of the key architectural enhancements. https://arxiv.org/abs/2410.17725.

[58] Tian Y., Ye Q., Doermann D. (2025). Yolov12: Attention-centric real-time object detectors. https://arxiv.org/abs/2502.12524.

[59] Lei M., Li S., Wu Y., Hu H., Zhou Y., Zheng X., Ding G., Du S., Wu Z., Gao Y. Yolov13: Real-time object detection with hypergraph-enhanced adaptive visual perception (2025). https://arxiv.org/abs/2506.17733.

[60] Lin X., Peng J., Gan Z., Zhu J., Liu J. (2026). Proceedings of the IEEE/CVF Conference on Computer Vision and Pattern Recognition (CVPR).

[61] Zhao Y., Lv W., Xu S., Wei J., Wang G., Dang Q., Liu Y., Chen J. (2024). Detrs beat yolos on real-time object detection. https://arxiv.org/abs/2304.08069.

[62] Yuan M., Wang D., Lin J., Yang S., Ning J. (2025). Ssp-mambanet: an automated system for detection and counting of missing seedlings in glass greenhouse-grown virus-free strawberry. Plant Phenomics.

[63] Xu B., Chen M., Guan W., Hu L. (2023). Efficient teacher: Semi-supervised object detection for yolov5. https://arxiv.org/abs/2302.07577.

[64] Yang Y., Ye R., Yin X., Tian H., Feng Z., Zhang Y., Yang J., Zhang X., Dong X., Tajima R. (2026). Seedling-detr: a detection transformer model for maize seedling monitoring using multispectral uav images. Remote Sens..

