# Supplementary material for: Label-efficient seedling perception via semi-supervised learning and counterfactual spatiotemporal feature rectification
Source: Plant Phenomics. 2026 Sep 8;8(4):100278. doi: 10.1016/j.plaphe.2026.100278 (PMC13720541; doi:10.1016/j.plaphe.2026.100278)
Supplement: Supplementary file 1 [file mmc1.pdf]

Table S1

Annotation volume and estimated active labeling effort under different sparse-anchor settings. Time estimates use 90 s per image, 8 s per bounding box, and an additional 20% allowance for review and correction.

Sparse-anchor ratio	Annotated boxes	Estimated active time (h)
10%	4,837	16.7
25%	11,886	40.8
50%	23,482	80.6
100%	46,676	160.1

Table S2

Seed-paired effects across the main comparative and ablation analyses. Values are reported as mean differences with two-sided 95% confidence intervals in percentage points.

Comparison	mAP@.50	mAP@[.5:.95]	Recall
Cross-architecture transfer (Δ = Ours − Base)			
RT-DETR-N	+0.66 [−0.26, 1.58]	+0.31 [−0.44, 1.06]	+1.97 [0.54, 3.40]
YOLOv8-S	+0.67 [0.20, 1.14]	+1.14 [0.43, 1.85]	+1.16 [0.26, 2.06]
YOLOv9-S	+0.56 [−0.63, 1.75]	+1.38 [0.26, 2.50]	+1.47 [−0.10, 3.04]
YOLOv10-S	+0.93 [0.49, 1.37]	+1.34 [0.95, 1.73]	+2.88 [1.76, 4.00]
YOLO11-S	+0.83 [0.61, 1.05]	+1.42 [1.08, 1.76]	+2.47 [1.69, 3.25]
YOLO12-S	+1.31 [0.60, 2.02]	+1.88 [1.14, 2.62]	+2.80 [2.01, 3.59]
YOLO13-S	+2.71 [0.94, 4.47]	+5.01 [1.03, 8.98]	+3.28 [1.16, 5.40]
YOLO-Master-S	+1.94 [1.47, 2.41]	+4.50 [3.96, 5.04]	+3.94 [2.98, 4.90]
YOLO12-S component ablation (Δ = setting − YOLO12-S)			
DART	+0.84 [−0.13, 1.81]	+0.86 [−0.35, 2.07]	+1.50 [0.39, 2.61]
MTI-Net	+1.10 [0.08, 2.12]	+1.52 [0.11, 2.93]	+2.25 [1.62, 2.88]
DART + MTI-Net	+1.31 [0.60, 2.02]	+1.88 [1.14, 2.62]	+2.80 [2.01, 3.59]
MTI-Net internal controls (Δ = MTI-Net − control)			
Temporal aggregation	+0.06 [−0.56, 0.68]	+0.41 [−0.53, 1.36]	+0.26 [−0.39, 0.91]
Random-box reconstruction	+0.40 [0.02, 0.77]	+0.70 [0.29, 1.11]	+0.14 [−0.71, 0.98]
DART stage-wise effects			
Bi-equal vs. Forward-only	+0.91 [−0.11, 1.93]	+1.11 [0.14, 2.07]	+0.14 [−0.89, 1.17]
Bi-uncertainty vs. Bi-equal	−0.16 [−2.17, 1.86]	+0.15 [−1.44, 1.74]	+0.83 [−0.01, 1.67]
Full DART vs. Bi-uncertainty	+0.62 [−1.11, 2.35]	+0.44 [−0.97, 1.86]	+0.43 [−0.17, 1.04]
Full DART vs. Forward-only	+1.37 [−0.08, 2.83]	+1.71 [1.03, 2.38]	+1.41 [−0.06, 2.87]
DART-centered sparse-anchor comparisons (Δ = DART-only − reference)			
10%: Supervised YOLO12-S	+3.56 [0.18, 6.94]	+6.75 [4.33, 9.17]	+2.59 [0.93, 4.25]
10%: SMT-inspired	+4.79 [1.43, 8.15]	+7.45 [5.08, 9.82]	+3.39 [0.35, 6.43]
10%: Efficient Teacher	+3.88 [−0.67, 8.43]	+7.36 [3.08, 11.65]	+2.95 [−1.43, 7.34]
25%: Supervised YOLO12-S	+1.55 [−0.72, 3.83]	+2.27 [0.32, 4.21]	+1.84 [−0.31, 3.98]
25%: SMT-inspired	+1.48 [0.20, 2.76]	+1.85 [0.53, 3.17]	+1.16 [−0.23, 2.55]
25%: Efficient Teacher	+1.14 [−1.39, 3.67]	+1.92 [−0.56, 4.41]	+1.49 [−0.51, 3.48]
50%: Supervised YOLO12-S	+0.43 [−1.17, 2.03]	+1.56 [0.40, 2.73]	+0.44 [−2.23, 3.10]
50%: SMT-inspired	+2.67 [0.08, 5.26]	+3.35 [0.85, 5.86]	+0.67 [−1.56, 2.90]
50%: Efficient Teacher	+0.44 [−0.26, 1.15]	+2.33 [1.10, 3.55]	+0.17 [−1.27, 1.61]

Table S3

DS-IM performance under different one-to-one Hungarian matching protocols. Precision, Recall, and F1-score are reported in %.

Matching criterion	TP	FP	FN	Precision	Recall	F1-score
Center-in-GT	183	28	39	86.73	82.43	84.53
IoU ≥ 0.3	184	27	38	87.20	82.88	84.99
IoU ≥ 0.5	174	37	48	82.46	78.38	80.37

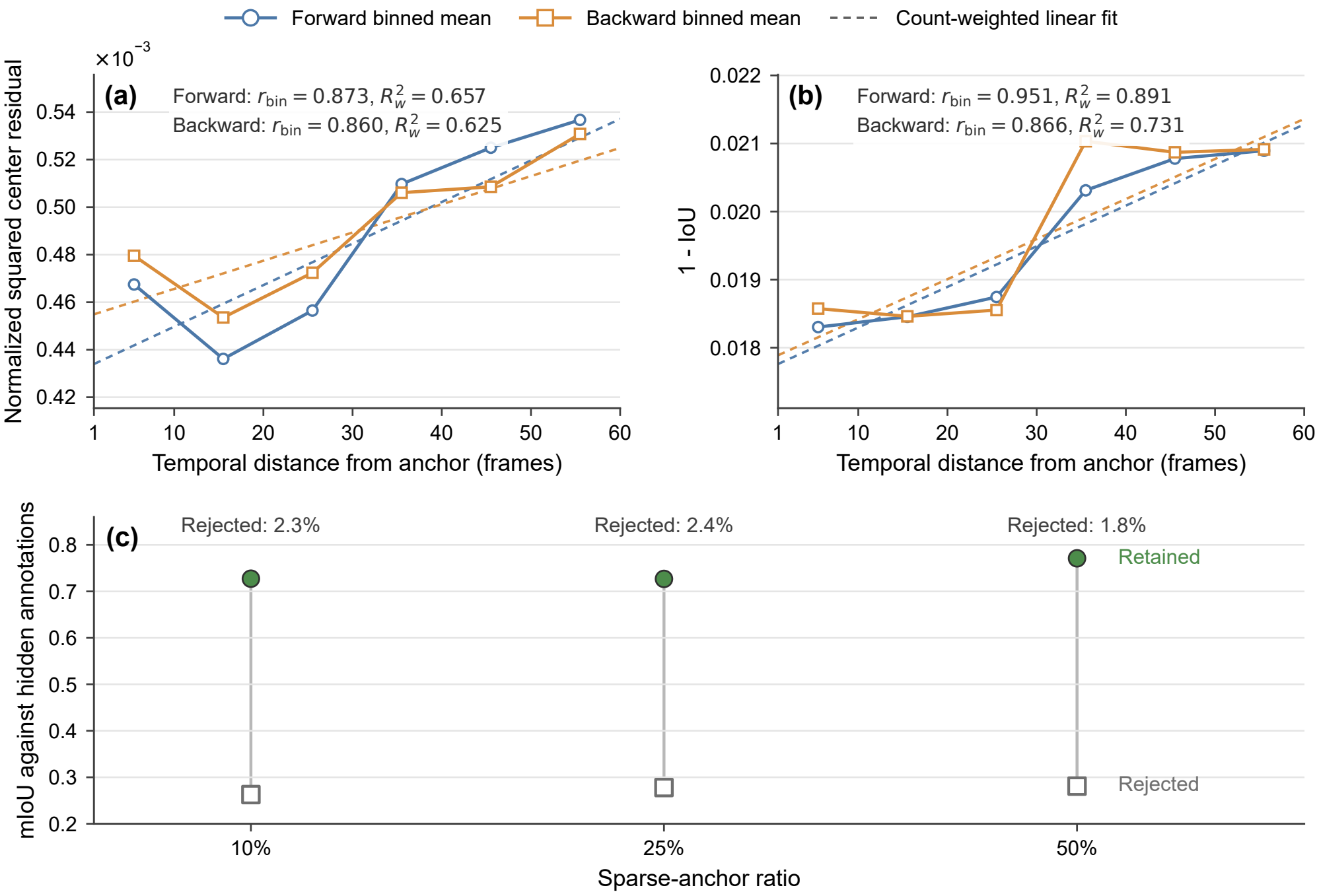

Figure S1: Empirical validation of the DART uncertainty mechanism. Panels (a) and (b) show the relationship between temporal propagation distance and tracking error; panel (c) compares the hidden-annotation mIoU of retained and rejected predictions across the evaluated sparse-anchor ratios. Dashed lines denote count-weighted least-squares fits.

Table S4

Frame-level gap-presence and count-evaluation results of DS-IM.

Gap presence		Gap count	
Metric	Value	Metric	Value
Precision	91.43%	MAE	0.229 gaps/frame
Recall	93.20%	RMSE	0.556 gaps/frame
F1-score	92.31%	Bias	−0.049 gaps/frame
Specificity	92.50%	Within ±1 gap	96.86%

Table S5

Matched comparison with semi-supervised detection baselines under different sparse-anchor ratios. All methods use the same YOLO12-S backbone, labeled partitions, training schedule, and five random seeds. Efficient Teacher, SMT-inspired, and DART-only additionally share the same unlabeled-frame pool.

Sparse-anchor ratio	Method	mAP@.50 (%)	mAP@[.5:.95] (%)	Recall (%)
10%	Supervised YOLO12-S	81.75 ± 1.88	48.31 ± 0.97	75.23 ± 1.06
	SMT-inspired	80.52 ± 1.03	47.61 ± 1.14	74.42 ± 1.15
	Efficient Teacher	81.43 ± 1.71	47.71 ± 1.59	74.80 ± 1.72
	DART-only	**85.30 ± 2.34**	**55.07 ± 2.12**	**77.81 ± 1.84**
25%	Supervised YOLO12-S	87.36 ± 1.34	58.05 ± 1.05	79.52 ± 1.48
	SMT-inspired	87.43 ± 1.62	58.47 ± 1.62	80.20 ± 1.51
	Efficient Teacher	87.77 ± 1.56	58.40 ± 1.72	79.88 ± 1.55
	DART-only	**88.91 ± 1.04**	**60.32 ± 0.74**	**81.36 ± 0.86**
50%	Supervised YOLO12-S	90.57 ± 1.00	64.12 ± 0.79	82.63 ± 1.54
	SMT-inspired	88.34 ± 1.74	62.33 ± 1.57	82.40 ± 1.29
	Efficient Teacher	90.56 ± 0.41	63.34 ± 0.65	82.89 ± 0.60
	DART-only	**91.01 ± 0.45**	**65.68 ± 0.69**	**83.06 ± 0.73**

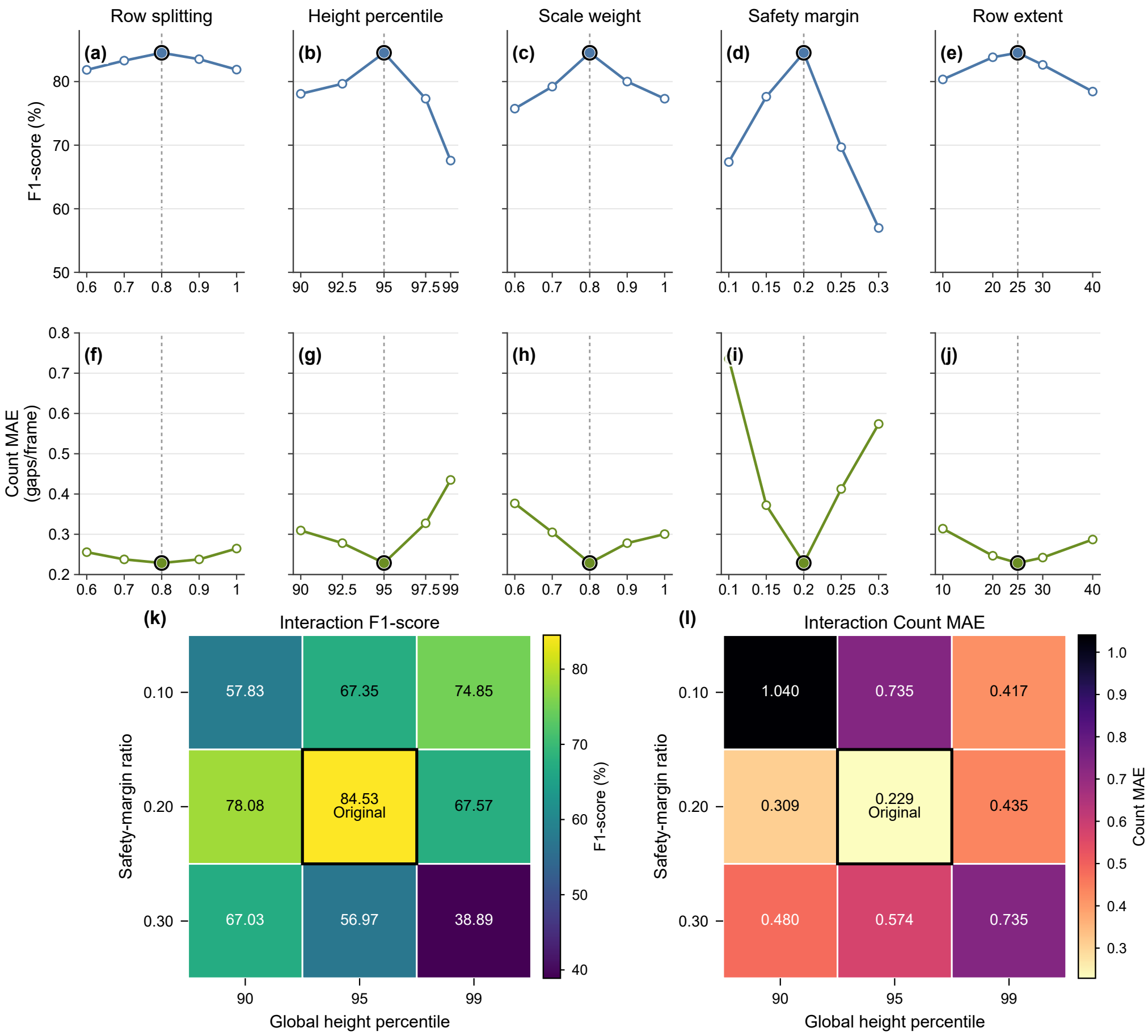

Figure S2: Sensitivity analysis of DS-IM parameters. Panels (a–j) show the effects of individual parameters on F1-score and count MAE, while panels (k,l) show the interaction between the safety margin and global height percentile.

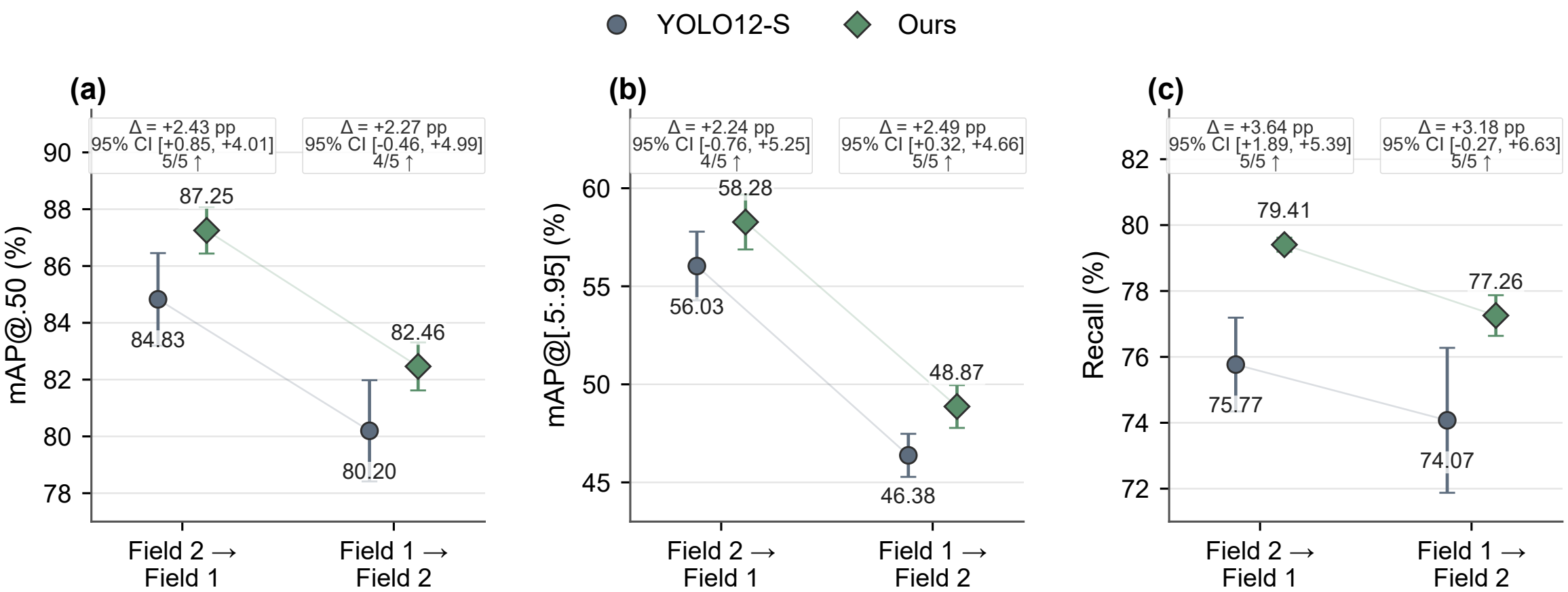

Figure S3: Leave-one-field-out applicability on YOLO12-S. Results are reported as mean ± standard deviation over five random seeds for the two cross-field transfer directions.

Table S6
Stage-wise ablations of DART and DS-IM. DART results are averaged over five random seeds, whereas DS-IM is evaluated deterministically on the wheat test set.

(a) DART under 25% sparse-anchor supervision

Setting	mAP@.50 (%)	mAP@[.5:.95] (%)	Recall (%)
Forward-only	87.54 ± 0.20	58.61 ± 0.44	79.95 ± 0.64
Bi-equal	88.45 ± 0.76	59.72 ± 0.98	80.09 ± 0.62
Bi-uncertainty	88.29 ± 1.27	59.88 ± 0.93	80.93 ± 1.05
Full DART	$\mathbf{88.91 \pm 1.04}$	$\mathbf{60.32 \pm 0.74}$	$\mathbf{81.36 \pm 0.86}$

(b) DS-IM on the wheat test set

Setting	Precision (%)	Recall (%)	F1 (%)	Count MAE (gaps/frame)
Macro reconstruction	31.78	79.73	45.44	1.637
+ Row-trajectory verification	38.71	75.68	51.22	1.103
+ Growth-space verification	**86.73**	**82.43**	**84.53**	**0.229**

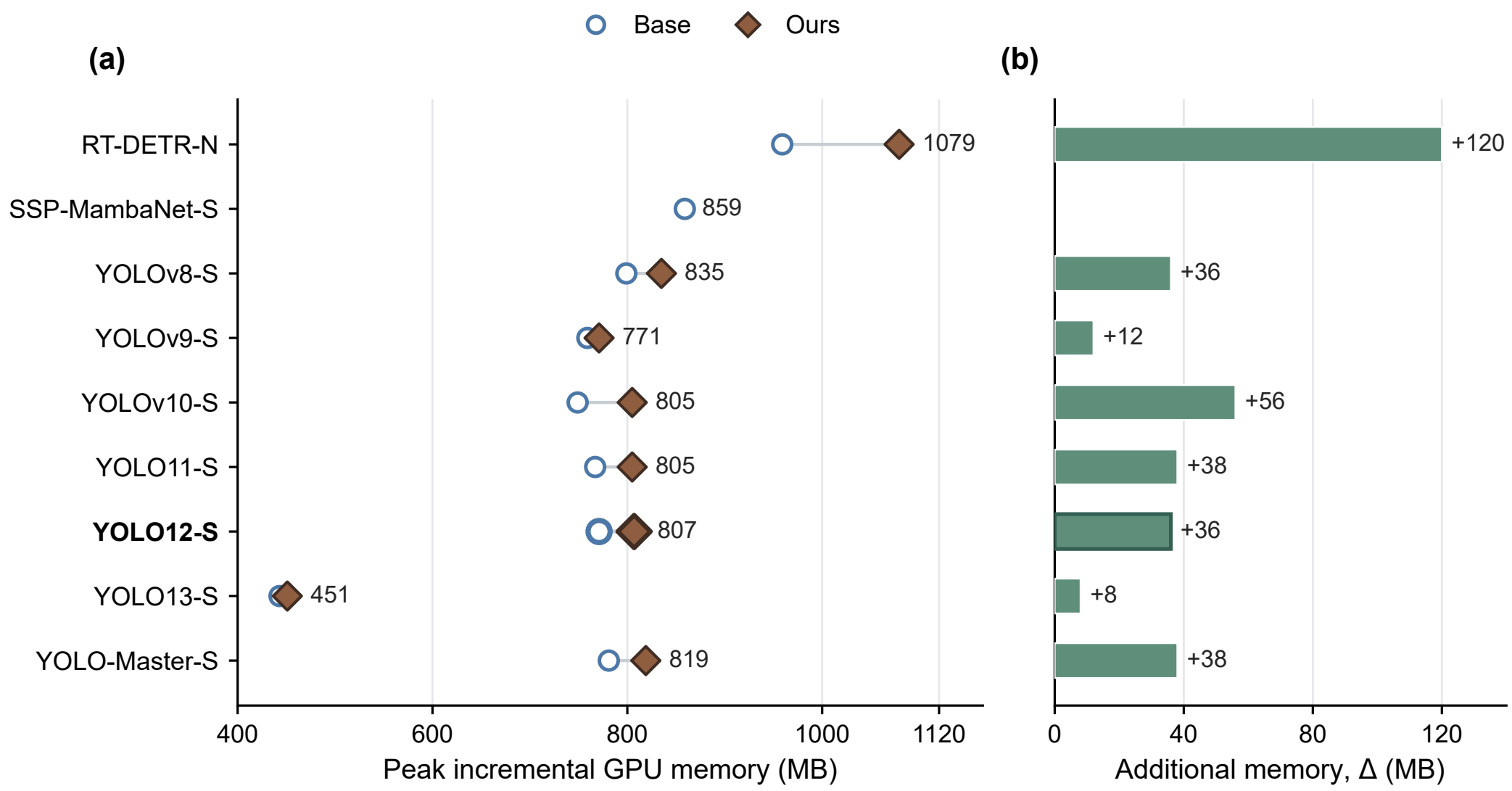

Figure S4: Peak incremental GPU memory across different detection architectures under batch-1 CUDA inference. Panels (a) and (b) show the measured peak incremental memory and the corresponding overhead, respectively.

Table S7
Glossary of principal mathematical symbols used in the proposed framework.

Symbol	Definition
t, t_{start}, t_{end}	Current frame and bounding temporal anchors
ΔT, Δt_{fwd}, Δt_{bwd}	Anchor interval and forward/backward propagation distances
$\mathcal{Z}^{(t)}_{fwd}$, $\mathcal{Z}^{(t)}_{bwd}$	Forward and backward tracking observations at frame t
$\mathcal{U}(t)$, τ_{unc}	Bidirectional disagreement uncertainty and shunting threshold
$\mathbf{x}^{(t)}$, $\hat{\mathbf{x}}^{(t)}$, $\mathbf{p}^{(t)}_k$	Latent state, DART-refined pseudo-label, and detector prediction
X, $\mathcal{X}_{hist}$	Backbone feature sequence and historical temporal features
$F^{(t)}_{cur}$, $\mathbf{M}$	Current reconstruction target feature and object-aware mask
$\tilde{F}^{(t)}$, $\hat{F}^{(t)}_{rec}$	Corrupted and reconstructed features
$\mathcal{L}_{det}$, $\mathcal{L}_{rec}$, $\mathcal{L}_{total}$, λ_{rec}	Detection, reconstruction, and total objectives and reconstruction weight
ΔY_{base}, N_{miss}	Baseline row spacing and estimated missing-row count
b_{virt}, δ_r	Virtual seedling hypothesis and adaptive growth-space margin
